# Diagnostics of Tuberculosis with Single-Walled Carbon Nanotube-Based Field-Effect Transistors

**DOI:** 10.1021/acssensors.3c02694

**Published:** 2024-03-14

**Authors:** Jieyu Wang, Wenting Shao, Zhengru Liu, Ganesh Kesavan, Zidao Zeng, Michael R. Shurin, Alexander Star

**Affiliations:** † Department of Chemistry, University of Pittsburgh, Pittsburgh, Pennsylvania 15260, United States; ‡ Department of Pathology, University of Pittsburgh Medical Center, Pittsburgh, Pennsylvania 15213, United States; § Department of Bioengineering, University of Pittsburgh, Pittsburgh, Pennsylvania 15261, United States

**Keywords:** Mycobacterium tuberculosis, diagnosis, rapid antigen test, antibody, biosensor

## Abstract

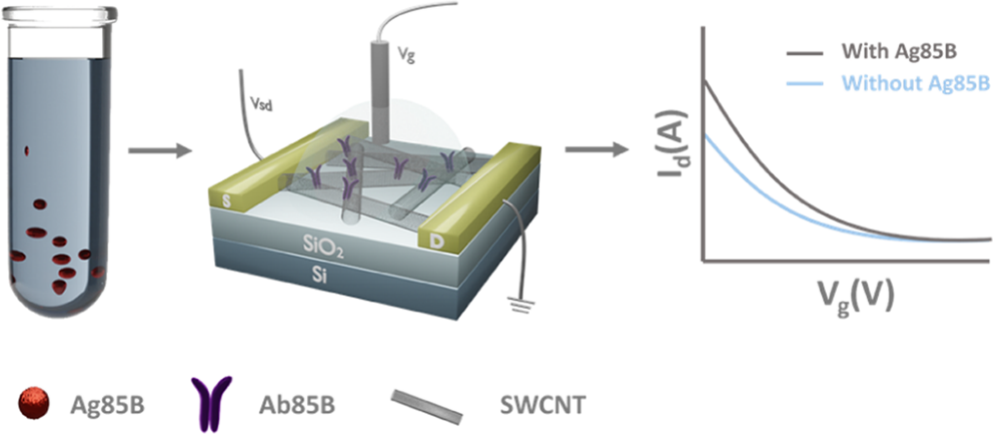

Tuberculosis (TB) is still threatening millions of people’s lives, especially in developing countries. One of the major factors contributing to the ongoing epidemic of TB is the lack of a fast, efficient, and inexpensive diagnostic strategy. In this work, we developed a semiconducting single-walled carbon nanotube (SWCNT)-based field-effect transistor (FET) device functionalized with anti-*Mycobacterium tuberculosis* antigen 85B antibody (Ab85B) to detect the major *M. tuberculosis*-secreted antigen 85B (Ag85B). Through optimizing the device fabrication process by evaluating the mass of the antibody and the concentration of the gating electrolyte, our Ab85B-SWCNT FET devices achieved the detection of the Ag85B spiked in phosphate-buffered saline (calibration samples) with a limit of detection (LOD) of 0.05 fg/mL. This SWCNT FET biosensor also showed good sensing performance in biological matrices including artificial sputum and can identify Ag85B in serum after introducing bovine serum albumin (BSA) into the blocking layer. Furthermore, our BSA-blocked Ab85B-SWCNT FET devices can distinguish between TB-positive and -negative clinical samples, promising the application of SWCNT FET devices in point-of-care TB diagnostics. Moreover, the robustness of this SWCNT-based biosensor to the TB diagnosis in blood serum was enhanced by blocking SWCNT devices directly with a glutaraldehyde cross-linked BSA layer, enabling future applications of these SWCNT-based biosensors in clinical testing.

Tuberculosis (TB), caused by *Mycobacterium tuberculosis* (MTB), is a highly contagious and airborne disease spread through the air when a patient coughs, speaks, or sneezes.^1^ According to the World Health Organization, MTB is one of the infectious agents that kill the most people in the world, and TB remains a leading cause of morbidity and mortality in many developing countries.^2,3^ TB is clinically dichotomized into active TB and latent TB forms.^4^ Latent TB is the case where the concentration of MTB is too low to show symptoms. The latest data from the Centers for Disease Control and Prevention show that there are 13 million people with latent TB in the USA, who, if left untreated, can turn into active TB. Thus, the early diagnosis of TB is essential in preventing its burden.

Currently, TB diagnosis focuses on screening tools (i.e., chest X-ray), the detection of bacilli by microscopic techniques (i.e., smear microscopy) and bacterial growth cultures, detection of host immune response to the pathogens in the skin (i.e., Mantoux or Pirquet test), and bacterial nucleic acid amplification methods.^5−7^ The interferon-γ release assays like QuantiFERON-TB Gold Plus, widely used in the USA and Europe, can also diagnose TB by detecting IFN-γ secretion from the collected blood lymphocytes after their stimulation with ESAT-6 and CFP-10 antigens that are quite specific for *M. tuberculosis*.^8^ Among them, a chest X-ray shows low sensitivity to latent TB; the Mantoux test needs several days before result reading and the accuracy of it depends on previous vaccinations. Although the molecular and functional immunological assays have high specificity, sensitivity, and accuracy, they are time-consuming and require specialized equipment, reagents, and operators, making them inappropriate for developing regions and limiting their widespread use.

At the forefront of digital health technology, there is an urgent demand for a rapid, simple, and effective TB diagnostic method. Nanobiosensors, the analytical detection tools based on functional nanomaterials that transduce biological responses into measurable signals, have great potential to satisfy the above requirements and have been applied in TB diagnosis through the detection of MTB-specific DNA, cells, or antigens.^9,10^ Korri-Youssoufi’s group fabricated carbon nanotube- and nanowire polypyrrole (nw-Ppy)-based biosensors. Both sensors were functionalized with PAMAM dendrimers, ferrocenyl group, and DNA probe and successfully detected the MTB DNA in real samples by cyclic voltammetry (CV) and square wave voltammetry (SWV).^11,12^ Although DNA detection provided high specificity in TB diagnosis, the real TB samples needed pretreatment like amplification by polymerase chain reaction (PCR) before sensing, which is complex and time-consuming. Kahng et al. tried to develop an immune-resistive biosensor to screen the MTB cells and the MTB antigen MTP64 through the functionalization of carbon nanotubes with specific antibodies.^13^ The LODs were 10 CFU/mL for cells and 100 ng/mL for MPT64 in tongue swab samples within 30 min. This work promises the application of cells or antigens as the biomarker in TB screening but shows low sensitivity. Furthermore, when antibodies are used as receptors, antigens are more favorable than cells as biomarkers due to antigen–antibody-specific interactions, which can decrease the LOD and increase the specificity, motivating their use in TB diagnostics.^14,15^ For instance, Bakhori et al. developed a nanobiosensor fabricated with antibody and CdSe-ZnS quantum dots/silica nanoparticles/screen-printed carbon electrode-modified electrode to detect the CFP10-ESAT-6 antigen complex.^16^ The LOD was improved to 0.15 ng/mL by linking enzyme catalase to the electrode and measuring generated differential pulse voltammetry (DPV) currents.

Field-effect transistor (FET)-based biosensors are increasingly recognized as a promising type of biosensor for their ability to provide rapid, sensitive, label-free, and highly specific detection of analytes.^17^ In 2016, Saengdee et al. developed a FET-based immunosensor for the detection of antigen 85 complex B (Ag85B), which is the major MTB-secreted product and has a high potential for binding to anti-TB antibodies.^18−20^ Through the integration of the silicon nitride layer and glutaraldehyde, the monoclonal antibody against recombinant Ag85B protein was immobilized onto the biosensor for antigen detection, and the LOD was determined as 0.12 μg mL^–1^. Recently, Ma et al. built a silicon nanowire-based field-effect transistor (SiNW-FET) biosensing platform to detect the MTB.^21^ Based on the binding between Ag85B and anti-Ag85B antibody, their biosensor showed good sensitivity and obtained responses from sputum samples of TB patients. These studies demonstrated the feasibility of TB diagnosis through the detection of MTB antigen–antibody interactions using nanomaterial-based FET biosensors.

Since 1998, single-walled carbon nanotubes (SWCNTs) have been utilized to create FETs, showcasing outstanding performance in biosensing owing to their unique physical properties.^22,23^ With an averaged diameter of approximately 1 nm, SWCNTs are comparable in size to biomolecules. They also exhibit relatively low charge-carrier density and high intrinsic carrier mobility that are preferred in detecting electrostatic interactions and charge transfer during biological processes.^24,25^ In contrast to other FET functional nanomaterials, such as graphene, silicon nitride, and silicon nanowires, SWCNTs with their extremely small diameter can reduce gate leakage and demonstrate high conductivity, biocompatibility, charge mobility, and stability.^26−29^ Additionally, a variety of proteins have been reported to be attached onto the sidewalls of SWCNTs through noncovalent (e.g., π–π stacking or polymer wrapping) or covalent functionalization (e.g., fluorination of SWCNTs or protein coupling via carboxyl groups), making SWCNTs well-suited for biosensing.^30−32^ Several researchers have fabricated SWCNT FET biosensors for medical applications, including the detection of SARS-CoV-2 antigens, cancer exosomal miRNA, and Alzheimer’s disease biomarkers, demonstrating LOD comparable to that of sophisticated methods like nucleic acid amplification tests (NAATs) and enzyme-linked immunosorbent assay (ELISA).^33−35^

Herein, we present an anti-MTB antigen 85B antibody-functionalized SWCNT (Ab85B-SWCNT) FET device to detect the major MTB-secreted antigen 85B (Ag85B) in biological fluids. Fabricated from commercial semiconductor-enriched SWCNTs, our FET devices had a high on/off ratio of ∼10^4^. Taking advantage of the presence of carboxyl groups on the sidewalls of commercial SWCNTs, Ab85B was functionalized onto SWCNTs through EDC/Sulfo-NHS coupling. To obtain the optimal sensing result, the mass of the antibody to decoration and the ion concentration of the gating electrolyte were investigated with calibration samples. Our calibration plots demonstrated that the Ab85B-SWCNT FET device could successfully detect Ag85B spiked in phosphate-buffered saline (PBS) with an LOD of 0.05 fg/mL. As current TB diagnostic methods concentrate on detecting MTB in sputum and serum matrices, we evaluated the performance of our Ab85B-SWCNT FET devices in artificial sputum and serum samples spiked with varying concentrations of Ag85B. The device can identify the presence of Ag85B spiked in artificial sputum. Moreover, bovine serum albumin (BSA)-blocked Ab85B-SWCNT FET devices can detect Ag85B spiked in serum and can distinguish TB-positive clinical samples from negative samples under 10 min with portable Metrohm potentiostat, highlighting the potential practicality of our biosensor for TB diagnosis. The robustness of the Ab85B-SWCNT devices to biofluids was further enhanced by integrating a BSA cross-linking blocking layer onto SWCNT networks. The Ab85B-cBSA-SWCNT FET device can undergo 12 serum tests without the blocking layer being washed away. This approach will provide opportunities for the development of robust biosensors for medical diagnostics.

## Results and Discussion

### Characterization of Ab85B-SWCNT FET Devices

**Figure 1:**
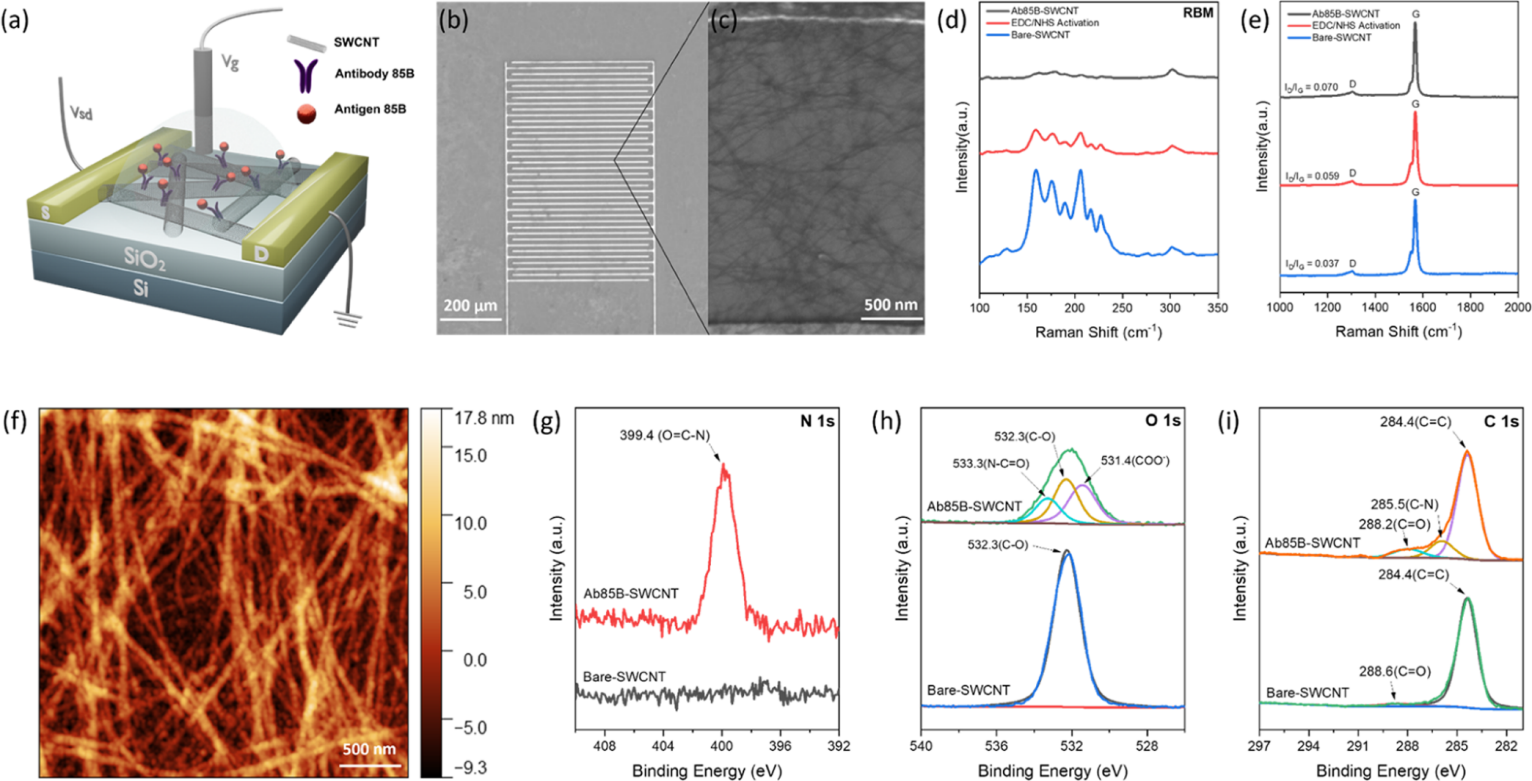
Characterization of Ab85B-SWCNT FET devices. (a) Schematic illustration of a Ab85B-SWCNT FET for detection of MTB antigen Ag85B. Interdigitated gold electrodes (yellow blocks) contacting a network of SWCNTs are configured as the source (S) and drain (D) electrodes. The source–drain voltage (*V*_sd_) is 50 mV. Gate voltage (*V*_g_) is applied through an Ag/AgCl reference electrode inserted into the gating electrolyte. (b) Scanning electron microscopy (SEM) image of a bare-SWCNT FET device. (c) SEM image of SWCNT network deposited between electrodes. (d) RBM region and (e) D and G peak regions of Raman spectra of the SWCNTs during functionalization. The RBM region was recorded using a 785 nm excitation laser. All spectra were normalized to the Si peak at 520 cm^–1^. D and G peak regions were recorded using a 638 nm excitation laser. All spectra were normalized to the G peak at 1569 cm^–1^. (f) AFM image of an Ab85B-SWCNT FET device. (g) High-resolution XPS spectra of N 1s of the bare-SWCNT and Ab85B-SWCNT FET device. (h) High-resolution XPS spectra of O 1s of the bare-SWCNT and Ab85B-SWCNT device, and (i) C 1s of the bare-SWCNT and Ab85B-SWCNT device with deconvolutions of the overall signal.

Anti-MTB Ag85B antibody-functionalized SWCNT (Ab85B-SWCNT) FET devices for specific detection of Ag85B protein were fabricated on silicon chips with gold interdigitated electrodes (IDEs) (Figure 1a). SWCNTs were deposited using dielectrophoresis (DEP) between the IDEs (Figure 1b). Scanning electron microscopy (SEM) imaging reveals the formation of interconnected networks of carbon nanotubes between the fingers of IDEs (Figure 1c). Through 1-ethyl-3-(3-(dimethylamino)propyl)carbodiimide/*N*-hydroxysulfosuccinimide (EDC/sulfo-NHS) coupling, carboxyl groups on SWCNTs were activated into amine-reactive O-acylisourea intermediates, where Ab85B was conjugated.^36^ Raman spectroscopy tracked the activation of carboxyl groups and functionalization of Ab85B proteins. Several peaks were observed in the radial breathing mode (RBM) region (150–250 cm^–1^), suggesting the diameter distribution of SWCNTs (Figure 1d).^37,38^ The decrease of peak intensity in the RBM region indicated the successful EDC/sulfo-NHS coupling and antibody conjugation because the functionalization broke the symmetry of SWCNT.^39^ The Raman spectra also featured the D peak (1302 cm^–1^) and G peak (1569 cm^–1^), and the ratio between the intensities of D and G peaks (*I*_D_/*I*_G_) reflected the defects on SWCNTs (Figure 1e).^37^ The observed increase of *I*_D_/*I*_G_ corresponded to the increase in defect degree caused by the antibody functionalization. The antibody decoration was also characterized by atomic force microscopy (AFM) (Figures 1f, S1, and S2). Based on the calculation of 20 AFM height profiles, the surface height increased by 4.05 nm after the decoration of Ab85B (Figures S3 and S4), indicating the successful conjugation of Ab85B to carbon nanotube sidewalls. X-ray photoelectron spectroscopy (XPS) has provided further evidence of Ab85B conjugation onto SWCNTs. After the immobilization of Ab85B onto SWCNTs, the O=C–N (399.4 eV) peak appeared in the XPS spectra of N 1s (Figure 1g). Before incorporating Ab85B onto SWCNT, the C–O (532.3 eV) peak in O 1s XPS spectra indicated the presence of defects on SWCNTs. After it, N=C–O at 533.3 eV, C–O at 532.3 eV, and COO^–^ at 531.4 eV can be identified in the Ab85B-SWCNT O 1s XPS spectra by deconvolution of a single broad peak, demonstrating the successful functionalization of carbon nanotubes with antibodies (Figure 1h).^40^ The C–N (285.5 eV) peak was also observed in the deconvoluted C 1s XPS spectra due to the introduction of Ab85B (Figures 1i and S5).

### Optimization of Device Functionalization

**Figure 2:**
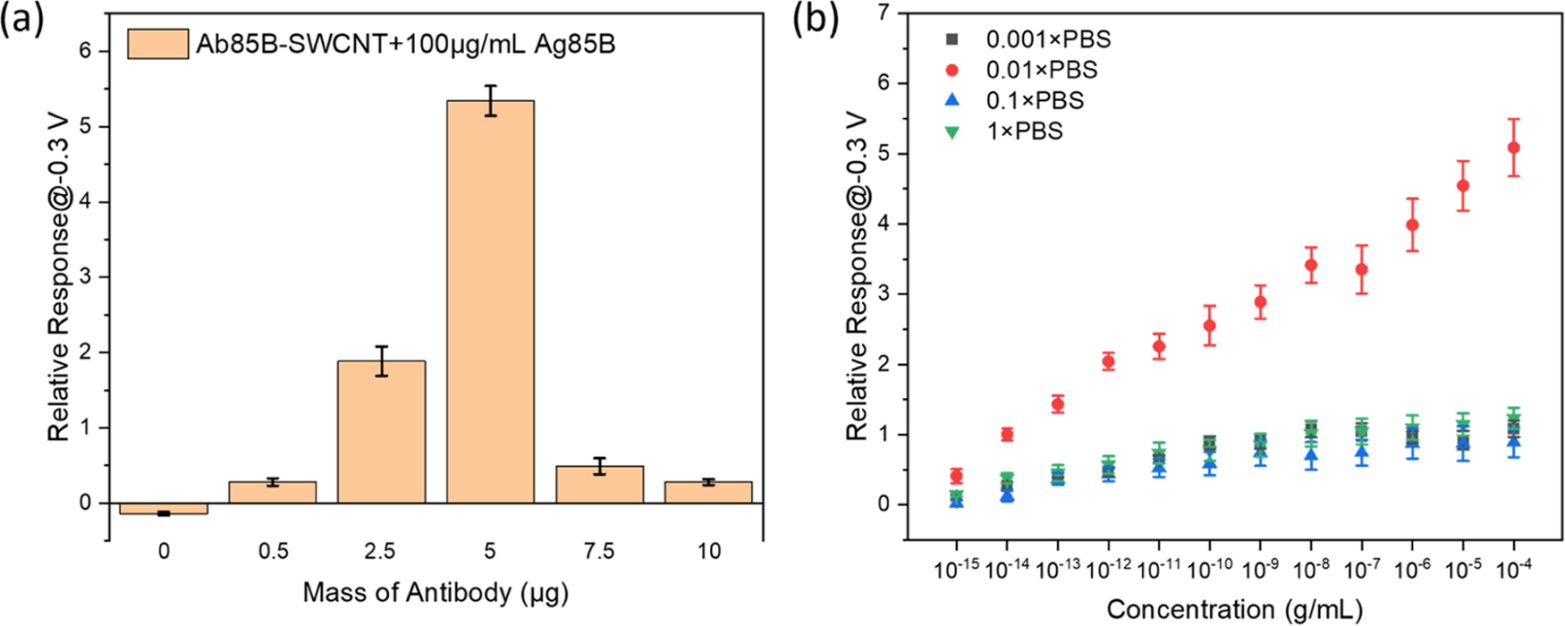
(a) Relative responses after adding 10 μL (100 μg/mL) of Ag85B solutions onto the SWCNT devices functionalized with different amounts of Ab85B; . All data points plotted in the histogram are mean ± standard error of the mean based on 3 devices. (b) Calibration plots showing the effect of the gating electrolyte on the Ag85B detection. All data points plotted in the calibration plots are mean ± standard error of the mean based on 5 devices.

One important factor that contributes to the sensitivity of SWCNT-based FET biosensors is the Debye screening length.^41^ The principle of Debye screening is stated in Supporting Information (SI). One strategy toward mitigating the Debye screening effect is to optimize the loading of Ab85B on the SWCNT surfaces so that the available binding sites can be maximized while keeping the height of the biorecognition layer within the Debye screening length. To accomplish this optimization step, we incubated activated bare-SWCNT devices with 0 (control), 0.5, 2.5, 5, 7.5, and 10 μg Ab85B for 12 h. After blocking, these devices were immersed in 10 μL of Ag85B solution (100 μg/mL in PBS) before FET measurements. The histogram of the relative response vs mass of Ab85B used is presented in Figure 2a. We observed that the response showed a rising trend when we added Ab85B from 0 to 5 μg, demonstrating a gradual saturation process for the loading of Ab85B on SWCNT devices. However, the response decreased from 534.1 to 27.88% when the mass of Ab85B was further added to 10 μg, implying the antibody–antigen interactions cannot be detected by SWCNT devices, which may be due to the formation of a new antibody configuration where the excess antibody proteins stayed nonspecifically on the device, leading to the biorecognition layer away from the Debye length. To prove the hypothesis, we performed FET measurements on SWCNT devices before and after the antibody functionalization. The calibration plot (Figure S6) showed a decreasing trend first (0–5 μg), which can be explained as the change of electronic properties of the metal–nanotube contact due to protein adsorption.^42^ Then, the response became relatively stable, proving the antibody loading saturation happened when 5 μg of Ab85B was introduced onto SWCNT devices. We further characterized SWCNT devices with different amounts of antibody by fluorescence microscopy. As Figure S7 shows, the fluorescence intensity of 10 μg-Ab85B SWCNT devices was higher than 5 μg-Ab85B SWCNT devices, indicating the existence of excess antibodies on the biosensor. Therefore, we chose to use the 5 μg Ab85B in the following antibody functionalization.

Liquid gating has been extensively utilized in our previous works as it is a favorable technique for biosensing, generating higher transconductance than back gating and reducing noise.^33,41,43−46^ The gating electrolytes of different concentrations have different ionic strength *I*, which thus give different Debye screening lengths, as stated in SI. Therefore, we explored the effect of a series of PBS solutions (1× to 0.001× PBS) as the gating electrolyte for the detection of Ag85B by Ab85B-SWCNT FET devices. The calibration plot showed that when using 0.01× PBS as the gating liquid, the relative response was significantly larger than other gating electrolytes (Figure 2b). Previous studies have concluded that the sensing responses optimized when the distance of the bound charges in receptor–ligand complexes to the SWCNT surface was within the Debye screening length.^21,47,48^ After the introduction of Ag85B, it would bind specifically to the Ab85B. Therefore, we could assume the height of bound charged species as the average height of the antibody (4.05 nm), which is within the Debye screening length of 0.01× PBS (7.4 nm) and 0.001× PBS (20 nm). However, the relative response of 0.001× PBS was not satisfactory, possibly because the low ion concentration is not favorable to the protein binding.^21^

### Detection of Ag85B in PBS

**Figure 3:**
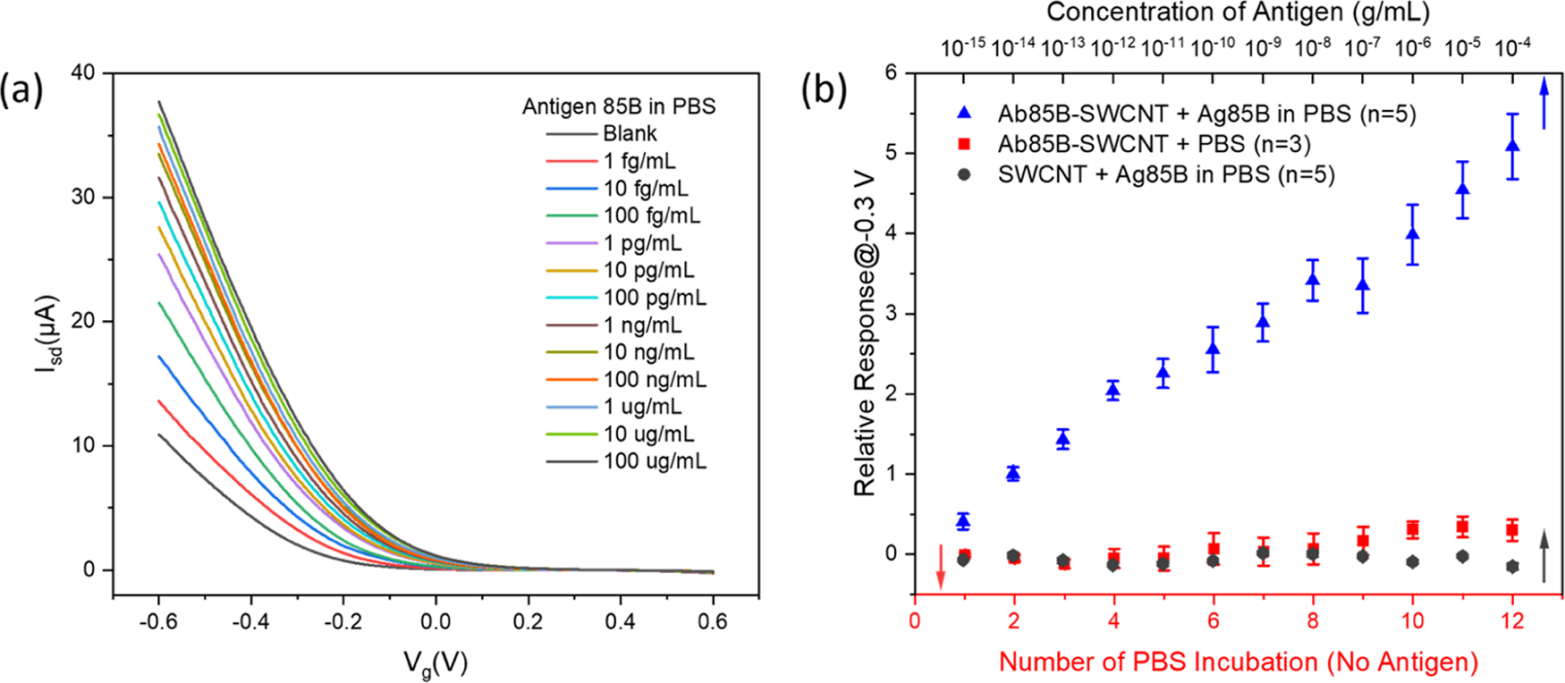
Detection of Ag85B protein in PBS. (a) FET characteristic curves of an Ab85B-SWCNT FET device upon exposure to increasing concentrations of Ag85B in PBS. (b) Calibration plot for Ag85B detection, effect from solvent and nonspecific binding detection. All data points plotted in the calibration plots are mean ± standard error of the mean. The number of devices (n) used is indicated in the parentheses.

After finding the optimal factors for device fabrication, we investigated the performance and sensing mechanism of the Ab85B-SWCNT FET devices for the detection of Ag85B proteins spiked in PBS as a calibration. Figure 3a shows the FET transfer characteristics, i.e., *I* – *V*_g_ curves, of an Ab85B-SWCNT FET device after exposure to Ag85B solutions (1 fg/mL to 100 μg/mL). With the increasing antigen concentration, the characteristic curves shifted to more positive gate voltages, the threshold voltage showed an increasing trend (Figure S8), and the curve’s linear region slope decreased (Figure S9). All of these changes can be explained as the adsorption of negatively charged Ag85B (pI = 5.5) onto SWCNTs, inducing positive charges, p-doping SWCNTs, and increasing the mobility.^49−52^ The calibration curve for the detection of Ag85B proteins spiked in PBS is depicted in Figure 3b. The calibration curve was linearly fit (*y* = 0.4066 × log(*x*) + 6.6724), and a linear correlation coefficient of 0.9804 was obtained (Figure S10), showing a large dynamic range of this Ab85B-SWCNT device. The calibration sensitivity, defined as the slope of the calibration curve, is 0.4066. Furthermore, nonspecific binding was investigated by examining the responses of bare-SWCNT FET devices to Ag85B spiked in PBS. The influence of solvent was also explored by incubating Ab85B-SWCNT FET devices with pure PBS 12 times. The extremely low relative responses of these two control experiments shown in Figure 3b proved that relative responses of the Ab85B-SWCNT FET devices upon exposure to Ag85B solution came from the specific binding between Ab85B and Ag85B. To determine the LOD of Ab85B-SWCNT FET devices, we repeated measuring the signal of a blank device for 20 times (Figure S11).^53^ Based on the signal that exceeds 3 times the noise response, the log scale of the LOD was log(*x*) = −16.3 (Figure S10). Therefore, the LOD was antilog(−16.3) = 5 × 10^–17^ or 0.05 fg/mL, which is lower than the immune–polymerase chain reaction (I-PCR) method.^54^

### Detection of Ag85B in Complex Matrices

**Figure 4:**
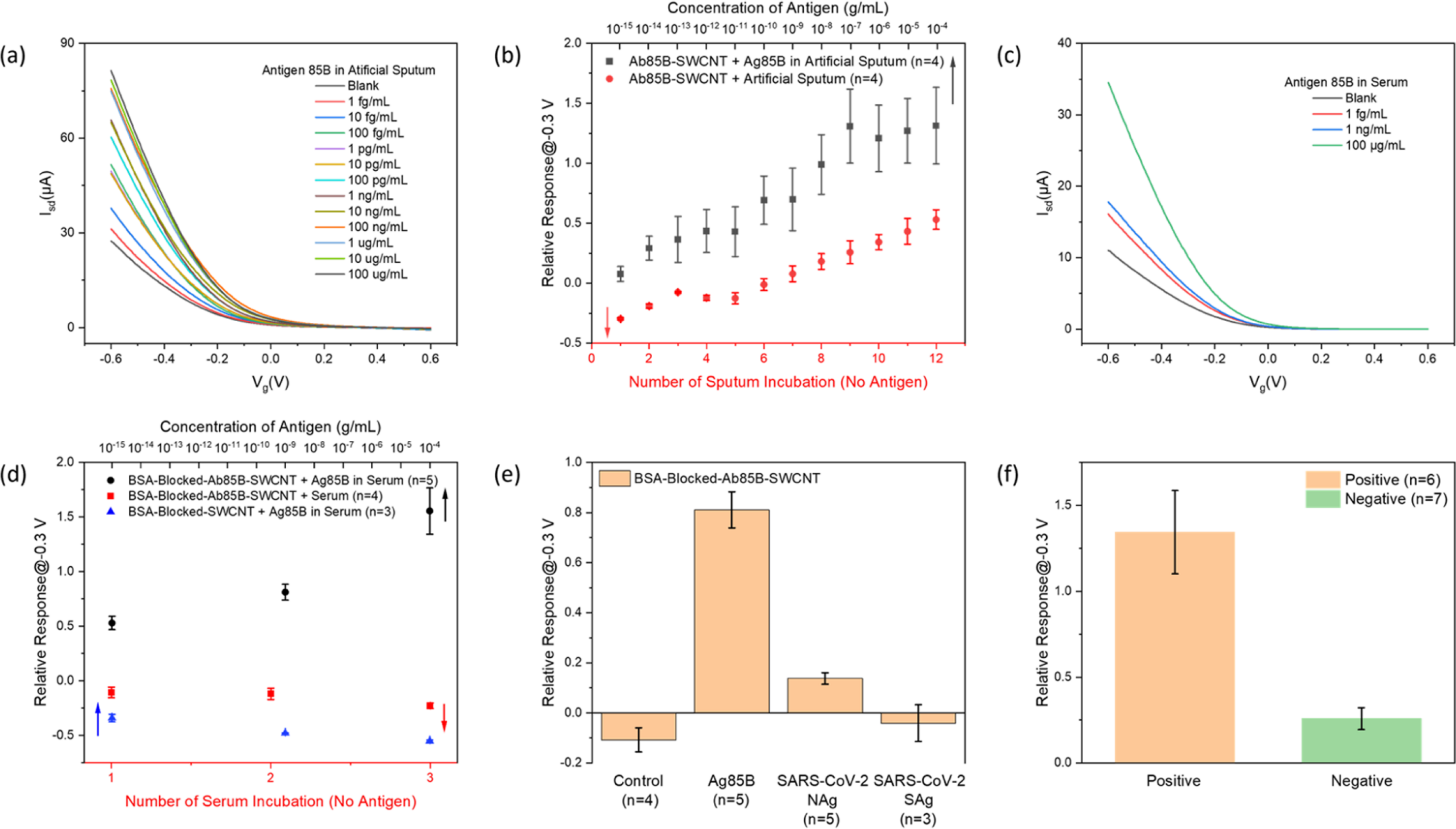
Detection of Ag85B protein in complex matrices. (a) FET characteristic curves of Ab85B-SWCNT FET devices upon exposure to increasing concentrations of Ag85B in artificial sputum. (b) Calibration plot showing the detection of Ag85B in artificial sputum and the corresponding control experiment studying the effect of artificial sputum. (c) FET characteristic curves of BSA-blocked-Ab85B-SWCNT FET devices upon exposure to increasing concentrations of Ag85B in human peripheral blood serum and (d) calibration plot showing the detection of Ag85B in serum and the corresponding control experiments studying the nonspecific interactions. (e) Specificity test of BSA-blocked-Ab85B-SWCNT FET devices. Control: pure serum (10 μL); experimental: Ag85B, SARS-CoV-2 NAg, and SARS-CoV-2 SAg (all proteins are 10 μL, 1 ng/mL in serum). (f) The relative responses after adding 10 μL of TB clinical blood samples onto the SWCNT FET devices. All data points plotted in the calibration plots are mean ± standard error of the mean and the number of devices (n) used is indicated in parentheses.

Our Ab85B-SWCNT FET devices were further studied to assess the detection of Ag85B proteins spiked in artificial sputum. The artificial sputum was prepared with 1 wt % methyl cellulose.^55^Figure 4a shows the *I* – *V*_g_ curves of the Ab85B-SWCNT FET device after exposure to Ag85B solutions in artificial sputum. Similar to the detection of Ag85B in PBS, a shift of the curves toward more positive gate voltage was observed, suggesting the successful detection of Ag85B proteins in artificial sputum. The calibration curve for the detection of Ag85B proteins in artificial sputum was plotted (Figure 4b) and further linearly fitted (Figure S12). Compared with the calibration plot of calibration samples, relatively larger error bars and smaller response change, calibration sensitivity (0.1197), and linear correlation coefficient (0.9683) were observed due to the effect of artificial sputum. Therefore, we further evaluated the performance of our Ab85B-SWCNT FET devices with the control experiment by exposing them to pure artificial sputum 12 times, and the calibration curve is plotted in Figure 4b. The difference of the relative responses between the experimental and control groups was observed from 1 fg/mL, implying that our devices could detect Ag85B at relatively low concentrations in artificial sputum samples. Despite the difference in responses, the increasing response from pure artificial sputum could not be ignored. The possible reason was that the negatively charged methyl cellulose (pI = 4.6) brought additional hole carriers to the SWCNTs; thus, the devices were p-doped due to electrostatic gating effect.^56^

We also tested our Ab85B-SWCNT FET device for the detection of Ag85B spiked in human peripheral blood serum (Figure S13). A control experiment examining the interference from serum to the biosensor was also performed. The response trend for detecting Ag85B in serum with Ab85B-SWCNT FET devices deviated from the trend for detecting Ag85B in PBS and overlapped with the control group trend, indicative of a compromised sensing capability toward Ag85B in human serum. This can be attributed to the nonspecific binding of serum components, such as albumin and globulin, to the device surface, causing interfering responses in the device, even though a blocking buffer with Tween 20 and PEG was applied. Therefore, the enhancement of the blocking buffer is essential for the test in the human serum.

To eliminate the influence of serum, we introduced serum, nonfat dry milk (NFDM), BSA, and BSA&NFDM into the Tween 20-polyethylene glycol (PEG) blocking buffer.^34,57^ To compare with the detection of Ag85B in PBS, we tested Ag85B in serum from 1 fg/mL to 100 μg/mL for each order of magnitude with Ab85B-SWCNT FET devices blocked by these four kinds of blocking buffers. The resulting calibration plot for detecting Ag85B protein in serum is presented in Figure S14. The negative relative response produced by serum-blocked Ab85B-SWCNT FET devices demonstrates the failure of Ag85B detection, which may be due to the excessive blocking by serum. The calibration plot for BSA-, NFDM-, and BSA&NFDM-blocked Ab85B-SWCNT FET devices exhibited the same increasing trend in the few concentration measurements at the beginning. However, then the calibration curves for all three kinds of devices showed a decreasing trend. This phenomenon could be attributed to the number of tests performed or other experimental factors. To obtain a calibration curve and explore the dynamic range, we typically test antigen solutions from 1 fg/mL to 100 μg/mL, covering each order of magnitude from low to high. In this experiment, we performed 12 tests to detect 12 different concentrations of Ag85B solutions. However, due to the incubation and washing process, which may cause the dissociation of NFDM or BSA and the recovery of serum influence, we observed a reduction in relative response after a certain number of tests. To test this hypothesis, we performed Ag85B detection from 1 fg/mL while reducing the number of tests to 6, 4, and 3 by adjusting the concentrations used in the detection with BSA-blocked-Ab85B-SWCNT FET devices. A control experiment testing the nonspecific bindings between the devices and serum was also performed. During the six-test experiment, we observed a decrease in relative response starting from the fifth test (100 ng/mL) as shown in Figure S15. Additionally, a difference in relative responses between the experimental and the control group was observed as early as 1 fg/mL. On the other hand, when we performed four tests, only an increasing trend of the relative response was observed, as shown in Figure S16. Based on this evidence, we can conclude that the decrease in relative response was not dependent on a specific concentration but rather related to the number of tests performed. To mitigate this influence, we decided to test the performance of our devices in detecting Ag85B in serum by evaluating three different concentrations (1 fg/mL, 1 ng/mL, and 100 μg/mL) using the BSA-blocked-Ab85B-SWCNT FET devices. As shown in Figure 4c, the *I* – *V*_g_ curves of the device after exposure to Ag85B in serum shifted toward a more positive gate voltage, similar to the detection of Ag85B in PBS, demonstrating the successful detection of Ag85B protein in serum. Figure 4d shows the calibration curve for the detection of Ag85B in serum along with the calibration curve depicting the effect of serum on our devices and the nonspecific binding between the antigen and electrode. The different trends in the experimental and control groups further demonstrated the feasibility of the BSA-blocked-Ab85B-SWCNT FET devices in detecting Ag85B in serum.

To further evaluate the specificity of BSA-blocked-Ab85B-SWCNT FET devices to Ag85B, an interference test was performed. The pure serum (10 μL) was incubated onto sensor chips for 10 min as the control group. Due to the Coronavirus disease 2019 (COVID-19) pandemic, severe acute respiratory syndrome coronavirus 2 (SARS-CoV-2) nucleocapsid protein (N antigen, NAg) and SARS-CoV-2 spike protein (S antigen, SAg) (10 μL, 10 ng/mL in serum) were chosen as related proteins for this specificity test. As Figure 4e shows, the relative responses from NAg and SAg were significantly lower than those from Ag85B, implying the high specificity of our BSA-blocked Ab85B-SWCNT devices to Ag85B.

### Detection of TB Clinical Samples

The BSA-blocked-Ab85B-SWCNT FET devices were further tested with TB clinical samples. One positive sample and one negative sample were tested separately with our potable TB detection device compositing of SWCNT FET devices and a Metrohm potentiostat (Figure S17). After the 10 min sample incubation, the device can give the result in several seconds. Figure 4f shows the relative response of the biosensor for the detection of TB-positive and -negative clinical samples. The histogram demonstrates that our portable biosensor can distinguish between the TB-positive and -negative samples successfully with a *p*-value of 7.0446 × 10^–4^ (α = 0.05) and has the potential in TB POC diagnosis.

### Enhancement of SWCNT Device Robustness

**Figure 5:**
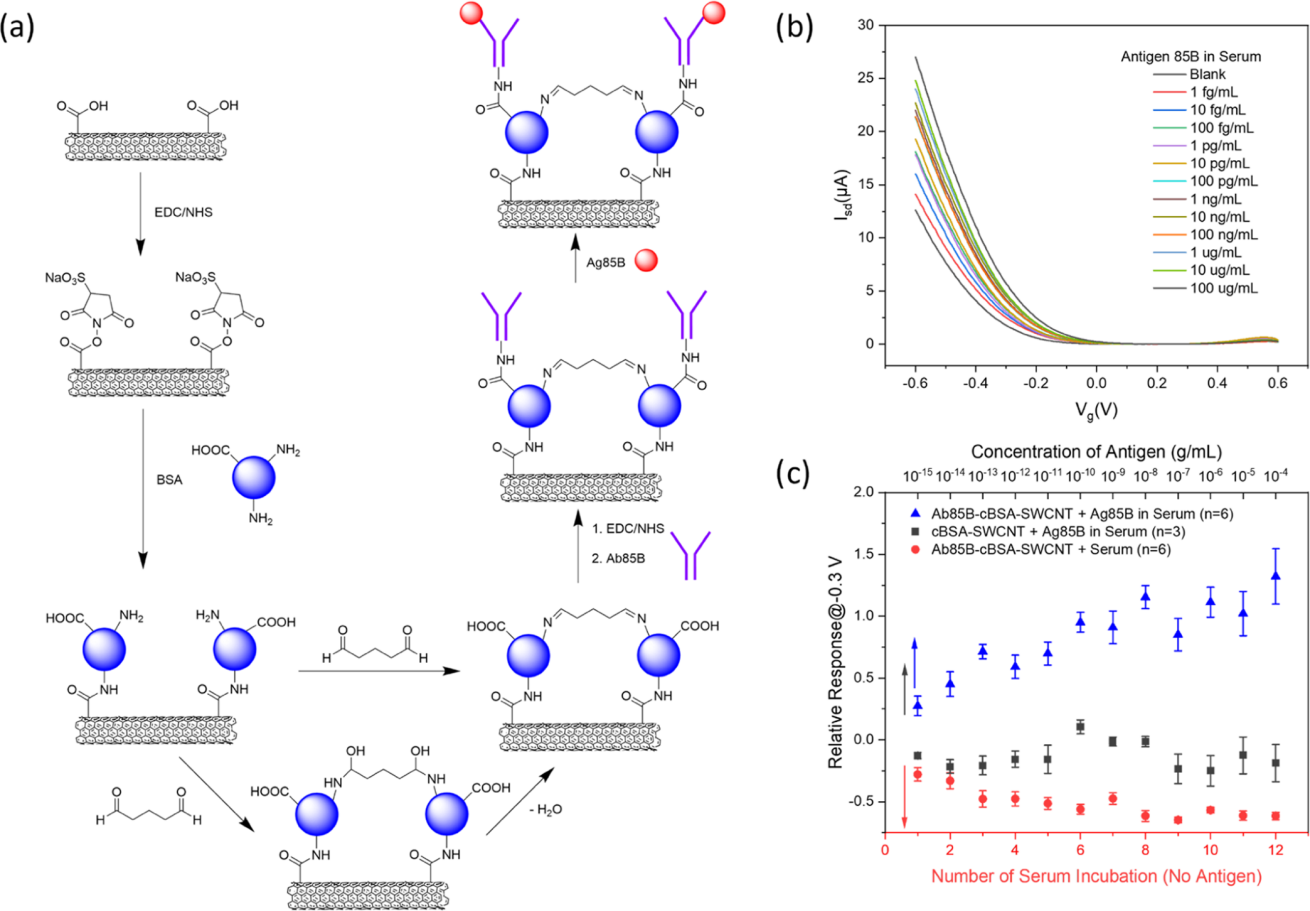
Detection of Ag85B protein in human peripheral blood serum with Ab85B-cBSA-SWCNT FET devices. (a) Schematic illustration of device surface functionalization process. (b) FET characteristic curves of an Ab85B-cBSA-SWCNT FET device upon exposure to increasing concentrations of Ag85B in serum. (c) Calibration plot for Ag85B detection, effect from serum and nonspecific binding detection. All data points plotted in the calibration plots are mean ± standard error of the mean. The number of devices (n) used is indicated in the parentheses.

To promote our biosensors in clinical diagnosis, the robustness of the devices to biofluids and the stability of the blocking layer should be improved (i.e., blocking layer binding tightly to devices and hard to be washed away). Previous work has demonstrated that the mixture of nanotube/BSA/glutaraldehyde (GA) can be a stable and antifouling biosensor coating layer due to the formation of a thick and porous BSA matrix around the nanotube, which could greatly reduce the nonspecific binding at the same time.^58^ Inspired by it, we tried to bind BSA directly onto the SWCNTs and then cross-link BSA with GA. Balavoine et al. demonstrated that the streptavidin proteins could adsorb on CNTs through noncovalent hydrophobic interactions.^59^ Therefore, the SWCNT FET device was first blocked with a BSA cross-linking layer noncovalently for Ag85B detection. The results are shown in Figure S18, and the variation in response trend could still be seen after a few tests, implying the weak affinity between SWCNTs and BSA proteins. Thus, we decided to bind the BSA cross-linking layer to SWCNTs covalently through EDC/Sulfo-NHS coupling. The surface functionalization process of the Ab85B functionalized-cross-linked BSA-blocked-SWCNT (Ab85B-cBSA-SWCNT) FET devices is shown in Figure 5a. SEM imaging characterized the process of BSA binding and cross-linking. Figure S19 shows the BSA proteins binding to SWCNT devices. After the introduction of GA to BSA, a dense layer covering SWCNT and aggregated particles was observed on the device surface (Figure S20), which was further characterized by fluorescence images. As Figure S21 shows, our devices exhibited green and red fluorescence, confirming the existence of a BSA cross-linking layer and some aggregated BSA protein.^60^

To evaluate the performance of our Ab85B-cBSA-SWCNT FET devices, we tested Ag85B in serum from 1 fg/mL to 100 μg/mL for each order of magnitude. Although the addition of the BSA cross-linking layer between SWCNT and Ab85B increases the height of the biorecognition layer, it also helps mitigate the Debye screening effect. Due to the high cost of entropy for ion partition from the solution to the cross-linking layer, the concentration of ions is significantly smaller at the interface between the cross-linking layer and the solution.^61,62^ Therefore, the Debye screening length is increased and is higher than the biorecognition layer. Thus, we still choose 0.01× PBS as the gating liquid. Figure 5b shows the *I* – *V*_g_ curves of an Ab85B-cBSA-SWCNT FET device after exposure to Ag85B solutions, and a shift of the curves toward more positive gate voltage was observed, suggesting the successful detection of Ag85B in serum. The corresponding calibration curve is plotted in Figure 5c and presents a single increasing trend similar to the detection in PBS. Compared with BSA-blocked Ab85B-SWCNT devices, the Ab85B-cBSA-SWCNT FET device can withstand 12 tests without the blocking layer being washed away, demonstrating the improved robustness of SWCNT-based biosensors and stronger evidence of the large dynamic range of SWCNT FET devices in detecting Ag85B in human serum. The effect of serum on the devices and nonspecific bindings between the cBSA-SWCNT device and Ag85B were also evaluated as control experiments and are plotted in Figure 5c. The deviation in the relative responses between the experimental and control groups can be observed from 1 fg/mL, indicating the increasing response trend coming from the antigen–antibody interactions and good sensitivity of the Ab85B-cBSA-SWCNT FET device. Further improvement of this approach would involve optimization of the ratio between SWCNT, GA, and BSA to maintain good robustness while minimizing the blocking layer thickness and maximizing the sensor response. Additionally, antibody fragments could be used instead of the whole antibody to minimize the distance between SWCNTs and the antigen binding site.

A comparison between our SWCNT FET biosensor device and previously reported sensor technologies for TB diagnosis is summarized in Table S1. This comparison shows that our SWCNT-based biosensor is more sensitive than most existing TB diagnostic methods. Notably, our biosensor can be used with both artificial sputum and blood serum and can identify the existence of Ag85B at extremely low concentrations (1 fg/mL). The blocking layer-enhanced SWCNT FET device can also recognize Ag85B in blood serum above 1 fg/mL. The large dynamic range is maintained even when exposed to biofluids. Additionally, the total test cost, including the sensor chip fabrication and antibody functionalization, is less expensive than present commercial TB tests.

## Conclusions

In conclusion, we fabricated an anti-*M. tuberculosis* antigen 85B antibody-functionalized SWCNT FET device for potential TB diagnostics. Through investigating the influence of the mass of antibody and gating electrolyte, the biosensor demonstrated high specificity and large dynamic range toward the detection of MTB-secreted Ag85B with an LOD of 0.05 fg/mL in calibration samples. We also tested the performance of the Ab85B-SWCNT FET device by detecting Ag85B spiked in complex matrices. The difference in relative responses between the experimental and control groups indicates that our devices have the ability to identify Ag85B in artificial sputum. Through the addition of BSA into the Tween 20-PEG blocking buffer, the Ab85B-SWCNT FET devices can detect Ag85B in serum and distinguish TB-positive clinical samples. The robustness of our SWCNT FET devices to serum samples was further improved by directly blocking the devices with a BSA cross-linking layer. In addition, the fabrication of each sensor is economical, and the test time is around 10 min, which indicates the possibility of developing a complete integrated point-of-care (POC) device with the antibody-functionalized SWCNT FET for the diagnosis of TB.

## Supporting Information Available

Experimental section; AFM images; height profiles; XPS spectra for SWCNT FET devices; threshold voltage and characteristic curve’s linear region slope change during Ag85B detection; calibration curves for detection of Ag85B in PBS; artificial sputum and serum with Ab85B-SWCNT devices; calibration curves comparing the blocking methods and studying the effect of the number of test on detecting Ag85B in serum; photo of portable detection device; SEM images of a BSA-SWCNT FET device and a cBSA-SWCNT FET device; fluorescence images of FITC-Ab85B SWCNT FET devices and cBSA-SWCNT FET devices (PDF).

## References

[ref1] Ridandari F.; Panjaitan A. C. Expert System to Diagnose Extra Lung Tuberculosis Using Bayes Theorem: Expert System to Diagnose Extra Lung Tuberculosis Using Bayes Theorem. J. Mantik 2019, 3 (3), 34–39.

[ref2] Chakaya J.; Khan M.; Ntoumi F.; Aklillu E.; Fatima R.; Mwaba P.; Kapata N.; Mfinanga S.; Hasnain S. E.; Katoto P. D.; Bulabula A. N.; Sam-Agudup N. A.; Nachega J. B.; Tiber S.; McHugh T. D.; Abubakar I.; Zumla A. Global Tuberculosis Report 2020–Reflections on the Global TB burden, treatment and prevention efforts. Int. J. Infect. Dis. 2021, 113, S7–S12. 10.1016/j.ijid.2021.02.107. 33716195 PMC8433257

[ref3] Gill C. M.; Dolan L.; Piggott L. M.; McLaughlin A. M. New developments in tuberculosis diagnosis and treatment. Breathe 2022, 18 (1), 210149 10.1183/20734735.0149-2021. 35284018 PMC8908854

[ref4] Blumberg H. M.; Ernst J. D. The challenge of latent TB infection. JAMA 2016, 316 (9), 931–933. 10.1001/jama.2016.11021. 27599327 PMC5319563

[ref5] Alnour T. M. Smear microscopy as a diagnostic tool of tuberculosis: Review of smear negative cases, frequency, risk factors, and prevention criteria. Indian J. Tuberc. 2018, 65 (3), 190–194. 10.1016/j.ijtb.2018.02.001. 29933859

[ref6] Simmons J. D.; Stein C. M.; Seshadri C.; Campo M.; Alter G.; Fortune S.; Schurr E.; Wallis R. S.; Churchyard G.; Mayanja-Kizza H.; Boom W. H.; Haw T. R. Immunological mechanisms of human resistance to persistent Mycobacterium tuberculosis infection. Nat. Rev. Immunol. 2018, 18 (9), 575–589. 10.1038/s41577-018-0025-3. 29895826 PMC6278832

[ref7] Acharya B.; Acharya A.; Gautam S.; Ghimire S. P.; Mishra G.; Parajuli N.; Sapkota B. Advances in diagnosis of Tuberculosis: an update into molecular diagnosis of Mycobacterium tuberculosis. Mol. Biol. Rep. 2020, 47, 4065–4075. 10.1007/s11033-020-05413-7. 32248381

[ref8] Dong B.; He Z.; Li Y.; Xu X.; Wang C.; Zeng J. Improved Conventional and New Approaches in the Diagnosis of Tuberculosis. Front. Microbiol. 2022, 13, 924410 10.3389/fmicb.2022.924410. 35711765 PMC9195135

[ref9] Bhalla N.; Pawan J.; Nello F.; Pedro E. Introduction to biosensors. Essays Biochem. 2016, 60 (1), 1–8. 10.1042/EBC20150001. 27365030 PMC4986445

[ref10] Carpenter A. C.; Paulsen I. T.; Williams T. C. Blueprints for biosensors: design, limitations, and applications. Genes 2018, 9 (8), 375 10.3390/genes9080375. 30050028 PMC6115959

[ref11] Miodek A.; Mejri N.; Gomgnimbou M.; Sola C.; Korri-Youssoufi H. E-DNA sensor of Mycobacterium tuberculosis based on electrochemical assembly of nanomaterials (MWCNTs/PPy/PAMAM). Anal. Chem. 2015, 87 (18), 9257–9264. 10.1021/acs.analchem.5b01761. 26313137

[ref12] Khoder R.; Korri-Youssoufi H. E-DNA biosensors of M. tuberculosis based on nanostructured polypyrrole. Mater. Sci. Eng.: C 2020, 108, 110371 10.1016/j.msec.2019.110371. 31924004

[ref13] Kahng S.-J.; Soelberg S. D.; Fondjo F.; Kim J.-H.; Furlong C. E.; Chung J.-H. Carbon nanotube-based thin-film resistive sensor for point-of-care screening of tuberculosis. Biomed. Microdevices 2020, 22, 50 10.1007/s10544-020-00506-3. 32725281

[ref14] Bahadır E. B.; Sezgintürk M. K. Applications of electrochemical immunosensors for early clinical diagnostics. Talanta 2015, 132, 162–174. 10.1016/j.talanta.2014.08.063. 25476294

[ref15] Yang X.; Fan S.; Ma Y.; Chen H.; Xu J.-F.; Pi J.; Wang W.; Chen G. Current progress of functional nanobiosensors for potential tuberculosis diagnosis: The novel way for TB control?. Front. Bioeng. Biotechnol. 2022, 10, 1036678 10.3389/fbioe.2022.1036678. 36588948 PMC9798010

[ref16] Bakhori N. M.; Yusof N. A.; Abdullah J.; Wasoh H.; Ab Rahman S. K.; Abd Rahman S. F. Surface enhanced CdSe/ZnS QD/SiNP electrochemical immunosensor for the detection of Mycobacterium tuberculosis by combination of CFP10-ESAT6 for better diagnostic specificity. Materials 2020, 13 (1), 149 10.3390/ma13010149. PMC6982155 31906075

[ref17] Shariati M. The field effect transistor DNA biosensor based on ITO nanowires in label-free hepatitis B virus detecting compatible with CMOS technology. Biosens. Bioelectron. 2018, 105, 58–64. 10.1016/j.bios.2018.01.022. 29355779

[ref18] Wiker H. G.; Harboe M. The antigen 85 complex: a major secretion product of Mycobacterium tuberculosis. Microbiol. Rev. 1992, 56 (4), 648–661. 10.1128/mr.56.4.648-661.1992. 1480113 PMC372892

[ref19] Ma Z.; Ji X.; Yang H.; He J.; Zhang Q.; Wang Y.; Wang Z.; Chen C. Screening and evaluation of Mycobacterium tuberculosis diagnostic antigens. Eur. J. Clin. Microbiol. Infect. Dis. 2020, 39, 1959–1970. 10.1007/s10096-020-03951-3. 32548683

[ref20] Saengdee P.; Chaisriratanakul W.; Bunjongpru W.; Sripumkhai W.; Srisuwan A.; Hruanun C.; Poyai A.; Phunpae P.; Pata S.; Jeamsaksiri W.; Kasinreak W.; Promptmas C. A silicon nitride ISFET based immunosensor for Ag85B detection of tuberculosis. Analyst 2016, 141 (20), 5767–5775. 10.1039/C6AN00568C. 27486595

[ref21] Ma J.; Du M.; Wang C.; Xie X.; Wang H.; Li T.; Chen S.; Zhang L.; Mao S.; Zhou X.; Wu M. Rapid and sensitive detection of mycobacterium tuberculosis by an enhanced nanobiosensor. ACS Sens. 2021, 6 (9), 3367–3376. 10.1021/acssensors.1c01227. 34470206

[ref22] Tans S. J.; Verschueren A. R.; Dekker C. Room-temperature transistor based on a single carbon nanotube. Nature 1998, 393 (6680), 49–52. 10.1038/29954.

[ref23] Martel R.; Schmidt T.; Shea H.; Hertel T.; Avouris P. Single-and multi-wall carbon nanotube field-effect transistors. Appl. Phys. Lett. 1998, 73 (17), 2447–2449. 10.1063/1.122477.

[ref24] Heller I.; Kong J.; Williams K. A.; Dekker C.; Lemay S. G. Electrochemistry at single-walled carbon nanotubes: the role of band structure and quantum capacitance. J. Am. Chem. Soc. 2006, 128 (22), 7353–7359. 10.1021/ja061212k. 16734491

[ref25] Allen B. L.; Kichambare P. D.; Star A. Carbon nanotube field-effect-transistor-based biosensors. Adv. Mater. 2007, 19 (11), 1439–1451. 10.1002/adma.200602043.

[ref26] Zou J.; Zhang Q. Advances and Frontiers in Single-Walled Carbon Nanotube Electronics. Adv. Sci. 2021, 8 (23), 2102860 10.1002/advs.202102860. PMC8655197 34687177

[ref27] Feigel I. M.; Vedala H.; Star A. Biosensors based on one-dimensional nanostructures. J. Mater. Chem. 2011, 21 (25), 8940–8954. 10.1039/c1jm10521c.

[ref28] Qadir A.; Pinke P.; Dusza J. Silicon Nitride-Based Composites with the Addition of CNTs—A Review of Recent Progress, Challenges, and Future Prospects. Materials 2020, 13 (12), 2799 10.3390/ma13122799. 32575905 PMC7345873

[ref29] Khan I.; Morshed O.; Mominuzzaman S. In A Comparative Performance Analysis of 10 nm Si Nanowire and Carbon Nanotube Field Effect Transistors, 17th International Conference on Nanotechnology (IEEE-NANO); IEEE, 2017; pp 109–112.

[ref30] Chen R. J.; Zhang Y.; Wang D.; Dai H. Noncovalent sidewall functionalization of single-walled carbon nanotubes for protein immobilization. J. Am. Chem. Soc. 2001, 123 (16), 3838–3839. 10.1021/ja010172b. 11457124

[ref31] Chen R. J.; Bangsaruntip S.; Drouvalakis K. A.; Kam N. W. S.; Shim M.; Li Y.; Kim W.; Utz P. J.; Dai H. Noncovalent functionalization of carbon nanotubes for highly specific electronic biosensors. Proc. Natl. Acad. Sci. U.S.A. 2003, 100 (9), 4984–4989. 10.1073/pnas.0837064100. 12697899 PMC154284

[ref32] Zhao Y.-L.; Stoddart J. F. Noncovalent functionalization of single-walled carbon nanotubes. Acc. Chem. Res. 2009, 42 (8), 1161–1171. 10.1021/ar900056z. 19462997

[ref33] Shao W.; Shurin M. R.; Wheeler S. E.; He X.; Star A. Rapid detection of SARS-CoV-2 antigens using high-purity semiconducting single-walled carbon nanotube-based field-effect transistors. ACS Appl. Mater. Interfaces 2021, 13 (8), 10321–10327. 10.1021/acsami.0c22589. 33596036

[ref34] Chen H.; Xiao M.; He J.; Zhang Y.; Liang Y.; Liu H.; Zhang Z. Aptamer-Functionalized Carbon Nanotube Field-Effect Transistor Biosensors for Alzheimer’s Disease Serum Biomarker Detection. ACS Sens. 2022, 7 (7), 2075–2083. 10.1021/acssensors.2c00967. 35816677

[ref35] Li T.; Liang Y.; Li J.; Yu Y.; Xiao M.-M.; Ni W.; Zhang Z.; Zhang G.-J. Carbon nanotube field-effect transistor biosensor for ultrasensitive and label-free detection of breast cancer exosomal miRNA21. Anal. Chem. 2021, 93 (46), 15501–15507. 10.1021/acs.analchem.1c03573. 34747596

[ref36] Fischer M. J. E. Amine Coupling through EDC/NHS: a Practical Approach. In Methods in Molecular Biology; Springer, 2010; Vol. 627, pp 55–73. 20217613 10.1007/978-1-60761-670-2_3

[ref37] Costa S.; Borowiak-Palen E.; Kruszynska M.; Bachmatiuk A.; Kalenczuk R. Characterization of carbon nanotubes by Raman spectroscopy. Mater. Sci.-Pol. 2008, 26 (2), 433–441.

[ref38] Dresselhaus M.; Jorio A.; Filho A. S.; Dresselhaus G.; Saito R. Raman spectroscopy on one isolated carbon nanotube. Phys. B 2002, 323 (1–4), 15–20. 10.1016/S0921-4526(02)00873-6.

[ref39] Dresselhaus M. S.; Dresselhaus G.; Saito R.; Jorio A. Raman spectroscopy of carbon nanotubes. Phys. Rep. 2005, 409 (2), 47–99. 10.1016/j.physrep.2004.10.006.

[ref40] Torres-González L.; Díaz-Ayala R.; Vega-Olivencia C. A.; López-Garriga J. Characterization of recombinant his-tag protein immobilized onto functionalized gold nanoparticles. Sensors 2018, 18 (12), 4262 10.3390/s18124262. 30518079 PMC6308469

[ref41] Shkodra B.; Petrelli M.; Angeli M. A. C.; Garoli D.; Nakatsuka N.; Lugli P.; Petti L. Electrolyte-gated carbon nanotube field-effect transistor-based biosensors: Principles and applications. Appl. Phys. Rev. 2021, 8 (4), 041325 10.1063/5.0058591.

[ref42] Chen R. J.; Choi H. C.; Bangsaruntip S.; Yenilmez E.; Tang X.; Wang Q.; Chang Y.-L.; Dai H. An investigation of the mechanisms of electronic sensing of protein adsorption on carbon nanotube devices. J. Am. Chem. Soc. 2004, 126 (5), 1563–1568. 10.1021/ja038702m. 14759216

[ref43] Lieb J.; Demontis V.; Prete D.; Ercolani D.; Zannier V.; Sorba L.; Ono S.; Beltram F.; Sacépé B.; Rossella F. Ionic-Liquid Gating of InAs Nanowire-Based Field-Effect Transistors. Adv. Funct. Mater. 2019, 29 (3), 1804378 10.1002/adfm.201804378.

[ref44] Liu Z.; Bian L.; Yeoman C. J.; Clifton G. D.; Ellington J. E.; Ellington-Lawrence R. D.; Borgogna J.-L. C.; Star A. Bacterial Vaginosis Monitoring with Carbon Nanotube Field-Effect Transistors. Anal. Chem. 2022, 94 (9), 3849–3857. 10.1021/acs.analchem.1c04755. 35191682

[ref45] Shao W.; Shurin G. V.; He X.; Zeng Z.; Shurin M. R.; Star A. Cerebrospinal fluid leak detection with a carbon nanotube-based field-effect transistor biosensing platform. ACS Appl. Mater. Interfaces 2022, 14 (1), 1684–1691. 10.1021/acsami.1c19120. 34932323

[ref46] Purwidyantri A.; Domingues T.; Borme J.; Guerreiro J. R.; Ipatov A.; Abreu C. M.; Martins M.; Alpuim P.; Prado M. Influence of the electrolyte salt concentration on DNA detection with graphene transistors. Biosensors 2021, 11 (1), 24 10.3390/bios11010024. 33477344 PMC7830926

[ref47] Stern E.; Wagner R.; Sigworth F. J.; Breaker R.; Fahmy T. M.; Reed M. A. Importance of the Debye screening length on nanowire field effect transistor sensors. Nano Lett. 2007, 7 (11), 3405–3409. 10.1021/nl071792z. 17914853 PMC2713684

[ref48] Vacic A.; Criscione J. M.; Rajan N. K.; Stern E.; Fahmy T. M.; Reed M. A. Determination of molecular configuration by Debye length modulation. J. Am. Chem. Soc. 2011, 133 (35), 13886–13889. 10.1021/ja205684a. 21815673

[ref49] Serafín-López J.; Talavera-Paulin M.; Amador-Molina J.; Alvarado-Riverón M.; Vilchis-Landeros M.; Méndez-Ortega P.; Fafutis-Morris M.; Paredes-Cervantes V.; Lopez-Santiago R.; León C.; Guerrero M.; Ribas-Aparicio R.; Mendoza-Hernández G.; Carreño-Martínez C.; Estrada-Parra S.; Estrada-García I. Enoyl-coenzyme A hydratase and antigen 85B of Mycobacterium habana are specifically recognized by antibodies in sera from leprosy patients. Clin. Vaccine Immunol. 2011, 18 (7), 1097–1103. 10.1128/CVI.00519-10. 21613461 PMC3147323

[ref50] Wahid A. A.; Doekhie A.; Sartbaeva A.; van den Elsen J. H. Ensilication improves the thermal stability of the tuberculosis antigen Ag85b and an Sbi-Ag85b vaccine conjugate. Sci. Rep. 2019, 9 (1), 11409 10.1038/s41598-019-47657-9. 31391509 PMC6685958

[ref51] Artyukhin A. B.; Stadermann M.; Friddle R. W.; Stroeve P.; Bakajin O.; Noy A. Controlled electrostatic gating of carbon nanotube FET devices. Nano Lett. 2006, 6 (9), 2080–2085. 10.1021/nl061343j. 16968029

[ref52] Heller I.; Janssens A. M.; Männik J.; Minot E. D.; Lemay S. G.; Dekker C. Identifying the mechanism of biosensing with carbon nanotube transistors. Nano Lett. 2008, 8 (2), 591–595. 10.1021/nl072996i. 18162002

[ref53] Bian L.; Shao W.; Liu Z.; Zeng Z.; Star A. Detection of Stress Hormone with Semiconducting Single-Walled Carbon Nanotube-Based Field-Effect Transistors. J. Electrochem. Soc. 2022, 169 (5), 057519 10.1149/1945-7111/ac6e8d.

[ref54] Singh N.; Sreenivas V.; Gupta K. B.; Chaudhary A.; Mittal A.; Varma-Basil M.; Prasad R.; Gakhar S. K.; Khuller G. K.; Mehta P. K. Diagnosis of pulmonary and extrapulmonary tuberculosis based on detection of mycobacterial antigen 85B by immuno-PCR. Diagn. Microbiol. Infect. Dis. 2015, 83 (4), 359–364. 10.1016/j.diagmicrobio.2015.08.015. 26422085

[ref55] Banik S.; Mahony J.; Selvaganapathy P. R. Elution of Artificial Sputum from Swab by Rotating Magnetic Field-Induced Mechanical Impingement. Appl. Sci. 2017, 7 (12), 1255 10.3390/app7121255.

[ref56] Cluskey F.; Thomas E.; Coulter S. Precipitation of milk proteins by sodium carboxymethylcellulose. J. Dairy Sci. 1969, 52 (8), 1181–1185. 10.3168/jds.S0022-0302(69)86721-4.

[ref57] Gaikwad P.; Rahman N.; Parikh R.; Crespo J.; Cohen Z.; Williams R. Optical nanosensor passivation enables highly sensitive detection of the inflammatory cytokine IL-6 bioRxiv 2023. 10.1021/acsami.4c02711 PMC11145596 38745465

[ref58] del Río J. S.; Henry O. Y.; Jolly P.; Ingber D. E. An antifouling coating that enables affinity-based electrochemical biosensing in complex biological fluids. Nat. Nanotechnol. 2019, 14 (12), 1143–1149. 10.1038/s41565-019-0566-z. 31712665

[ref59] Balavoine F.; Schultz P.; Richard C.; Mallouh V.; Ebbesen T. W.; Mioskowski C. Helical crystallization of proteins on carbon nanotubes: a first step towards the development of new biosensors. Angew. Chem., Int. Ed. 1999, 38 (13–14), 1912–1915. 10.1002/(SICI)1521-3773(19990712)38:13/14<1912::AID-ANIE1912>3.0.CO;2-2. 34182705

[ref60] Ma X.; Sun X.; Hargrove D.; Chen J.; Song D.; Dong Q.; Lu X.; Fan T.-H.; Fu Y.; Lei Y. A biocompatible and biodegradable protein hydrogel with green and red autofluorescence: preparation, characterization and in vivo biodegradation tracking and modeling. Sci. Rep. 2016, 6 (1), 19370 10.1038/srep19370. 26813916 PMC4728389

[ref61] Gao N.; Zhou W.; Jiang X.; Hong G.; Fu T.-M.; Lieber C. M. General strategy for biodetection in high ionic strength solutions using transistor-based nanoelectronic sensors. Nano Lett. 2015, 15 (3), 2143–2148. 10.1021/acs.nanolett.5b00133. 25664395 PMC4594804

[ref62] Piccinini E.; Alberti S.; Longo G. S.; Berninger T.; Breu J.; Dostalek J.; Azzaroni O.; Knoll W. Pushing the boundaries of interfacial sensitivity in graphene FET sensors: Polyelectrolyte multilayers strongly increase the Debye screening length. J. Phys. Chem. C 2018, 122 (18), 10181–10188. 10.1021/acs.jpcc.7b11128.

