# Supplementary material for: Diagnostics of Tuberculosis with Single-Walled Carbon Nanotube-Based Field-Effect Transistors
Source: ACS Sens. 2024 Mar 14;9(4):1957–66. doi: 10.1021/acssensors.3c02694 (PMC11059104; doi:10.1021/acssensors.3c02694)
Supplement: Supplementary file 1 — se3c02694_si_001.pdf [file se3c02694_si_001.pdf]

Supporting Information

Diagnostics of Tuberculosis with Single-Walled Carbon Nanotube-Based Field-Effect Transistors

Jieyu Wang,[†] Wenting Shao,[†] Zhengru Liu,[†] Ganesh Kesavan,[†] Zidao Zeng,[†] Michael R. Shurin[‡] and Alexander Star[*, †, §]

† Department of Chemistry, University of Pittsburgh, Pittsburgh, Pennsylvania 15260, United States

‡ Department of Pathology, University of Pittsburgh Medical Center, Pittsburgh, Pennsylvania 15213, United States

§ Department of Bioengineering, University of Pittsburgh, Pittsburgh, Pennsylvania 15261, United States

*Corresponding author. Email: astar@pitt.edu

Table of Contents

Experimental Section……………………………………………………...………………………S3

Principle of Debye Screening……………………………………………………………………...S9

Figure S1. AFM image of a bare-SWCNT FET device……………………………..…….….……S10

Figure S2. AFM image of an Ab85B-SWCNT FET device……………………………..….……..…..S10

Figure S3. Height profile of a bare-SWCNT FET device…………………………………..………....S11

Figure S4. Height profile of an Ab85B-SWCNT FET device…………………………………..…..…S11

Figure S5. XPS survey of a SWCNT FET device before and after Ab85B functionalization………...…...S12

Figure S6. Calibration plot for SWCNT FET devices after antibody functionalization…………………S12

Figure S7. Fluorescence characterization of FITC-Ab85B SWCNT FET devices……………..……..S13

Figure S8. Threshold voltage shift of Ab85B-SWCNT FET devices for Ag85B detection…..……..…..S14

Figure S9. Characteristic curve's linear region slope of Ab85B-SWCNT FET device……...……..…..S14

Figure S10. Calibration curve for detection of Ag85B in PBS with linear fitting…………..…...……..S15

Figure S11. FET characteristic curves for Ab85B-SWCNT FET device………...…...……………...……S16

Figure S12. Calibration curve for detection of Ag85B in artificial sputum with linear fitting ………..….S16

Figure S13. Calibration curves for detection of Ag85B in serum and serum control experiment by Ab85B-SWCNT FET devices…..……..…………………….…………………….………………..……..……..S17

Figure S14. Calibration curve for detection of Ag85B in serum by BSA&NFDM-, BSA-, NFDM-, and serum-blocked-Ab85B-SWCNT FET devices respectively……………...……………..……….…....S17

Figure S15. Calibration curve for detection of 6 different concentrations of Ag85B in serum by BSA-blocked-Ab85B-SWCNT FET devices……….……...…………………...………...…...……S18

Figure S16. Calibration curve for detection of 4 different concentrations of Ag85B in serum by BSA-blocked Ab85B-SWCNT FET devices………….……………………………….…………...…..S18

Figure S17. Photo showing the portable detection device with Metrohm potentiostat…………………...S19

Figure S18. Calibration plot for detection of Ag85B in human peripheral blood serum with Ab85B functionalized-BSA crosslinking layer non-covalently incubated-SWCNT FET devices………………S20

Figure S19. SEM image of a BSA-SWCNT FET device……………………………………………..S21

Figure S20. SEM image of a cBSA-SWCNT FET device……………………………………………….S21

Figure S21. Fluorescence image of cBSA-SWCNT FET devices ……………………………………….S22

Table S1. Comparison between different technologies in TB diagnosis………………………………S23

Experimental Section

1. Device Fabrication

By using photolithography, the interdigitated gold electrodes were patterned onto the 2.6 × 2.6 mm Si/SiO_2 wafers to form 6 μm channels. The chips were wire-bonded onto the standard 40-pin ceramic dual inline package and secured with polydimethylsiloxane (PDMS) by heating at 200 °C for 20 min. For the anti-MTB Ag85B antibody-functionalized SWCNT (Ab85B-SWCNT) FET devices, commercial semiconducting single-walled carbon nanotubes (SWCNTs) (IsoSol-S100, NanoIntegris) were used for the fabrication of sensors. 2 μL IsoSol-SWCNT (0.02 mg/mL in toluene) were deposited between gold electrodes by dielectrophoresis (DEP) with an applied bias voltage of 10 V and AC frequency of 100 kHz for 2 min. Isopropyl alcohol was used to wash excess IsoSol-SWCNT and the devices were annealed at 200 °C for 12 h before use. To functionalize anti-mycobacterium tuberculosis Ag85B antibody (Ab85B) onto the device, 1-ethyl-3-(3-dimethylaminopropyl)carbodiimide (EDC)/N-hydroxysulfosuccinimide (sulfo-NHS) coupling was used. Specifically, the devices were incubated with 100 μL of EDC/sulfo-NHS solution [50 mM/50 mM in phosphate-buffered saline (PBS)] for 30 min to activate the carboxyl groups on IsoSol-SWCNT into an amine reactive O-acylisourea intermediate. The excess EDC/sulfo-NHS solution was washed away with nanopure water. Then the devices were incubated with 10 μL Ab85B (500 μg/mL in PBS) (ab43019; Abcam, USA) for 12 h at 4°C. After a thorough washing with nanopure water, the devices were covered by a blocking buffer (0.1% Tween 20 and 4% polyethylene glycol in PBS) for 30 min to prevent unspecific interactions. The devices were washed with nanopure water again to remove any excess chemicals before the antigen 85B incubation and FET measurements.

2. FET Measurements

FET devices for the detection of Ag85B were investigated through liquid-gated FET device configuration by Keithley Source Meter Unit 2400. A 1 M Ag/AgCl electrode (CH Instruments, Inc.)

was used as the gate electrode and PBS was used as the gating electrolyte. In FET measurements, the gate voltage (V_g) was swept from +0.6 to −0.6 V with a source-drain voltage (V_{sd}) of 50 mV.

A series of recombinant mycobacterium tuberculosis Ag85B protein (ab83471; Abcam, USA) solutions from 1 fg/mL to 100 μg/mL were prepared with PBS. For each experiment, Ag85B solutions were tested from the lowest to the highest concentration. Specifically, the devices were incubated with 10 μL of the Ag85B solution for 10 min, which was then washed away with nanopure water thoroughly. Then the FET measurements were done with PBS solution as gating electrolyte.

The relative response of each FET device was calculated as $\Delta I/I_0$ at $V_g = -0.3$ V, where $\Delta I = I_{sd} - I_0$. I_{sd} is the source-drain current in PBS after antigen exposure and I_0 is the source-drain current in PBS before antigen exposure at $V_g = -0.3$ V. The figures were plotted with averaged relative responses of several devices with standard error of the mean as error bars. The number of devices (n) tested for each experiment was specified in the caption and figure.

For control experiments studying the effect of solvent, the FET devices were incubated with 10 μL of the solvent for 10 min, which was then washed away with nanopure water thoroughly. The FET measurements were done with PBS solution as gating electrolyte. This step was repeated 12 times since we studied antigen solutions with 12 orders of magnitude. The relative response was calculated as stated above.

3. Preparation of Artificial Sputum and Detection of Ag85B in Artificial Sputum

Artificial Sputum was prepared from methyl cellulose (Sigma-Aldrich, USA; Viscosity: 400 cP,). Specifically, 20 mL water was preheated to 80°C. The methyl cellulose (1 wt.%) was put into the hot water and the mixture was stirred until the particles were well dispersed. Then 40 mL water was poured into the beaker and the mixture was stirred for another half an hour to get transparent artificial sputum. The sputum could be stored at 4°C for 1 month. The artificial sputum was incubated onto Ab85B-SWCNT FET devices 12 times to examine the effect from artificial sputum. A series of Ag85B protein solutions from 1 fg/mL to 100 μg/mL were prepared with artificial sputum for antigen detection. Ag85B

solutions were tested from the lowest to the highest concentration and the FET measurement operation was the same as stated above.

4. Detection of Ag85B in Serum

Human serum (Fisher Scientific, USA, Cat# BP2657100) was used for this work. A series of Ag85B protein solutions from 1 fg/mL to 100 μg/mL were spiked in serum for antigen detection. Ab85B-SWCNT FET devices were first used for Ag85B detection, from the lowest to the highest concentration and the FET measurement operation was the same as stated above. The control experiment examining the interference from serum to the Ab85B-SWCNT FET device was performed by incubating serum onto sensor chip for 10 min, washing and performing detection with liquid gate, which was repeated 12 times.

To reduce the influence from serum, four kinds of blocking buffer were prepared to block the non-specific sites on the Ab85B-SWCNT devices respectively. Specifically, the serum blocking buffer was prepared with 0.1% Tween 20, 4% polyethylene glycol and 5% serum in PBS; the non-fat dry milk blocking buffer was prepared with 0.1% Tween 20, 4% polyethylene glycol and 5% NFDM in PBS; the BSA blocking buffer was prepared with 0.1% Tween 20, 4% polyethylene glycol and 1% BSA in PBS and the BSA&NFDM blocking buffer was prepared with with 0.1% Tween 20, 4% polyethylene glycol, 5% NFDM and 1% BSA in PBS. The devices were blocked by a buffer for 60 min. A series of Ag85B protein solutions from 1 fg/mL to 100 μg/mL were prepared in blood serum for antigen detection.

To prove the number of tests affecting the sensing result, detections for Ag85B in serum with different number of tests were performed. When testing twelve times, a series of Ag85B protein solutions in serum from 1 fg/mL to 100 μg/mL were tested by each order of magnitude; when testing six times, a series of Ag85B protein solutions in serum (1 fg/mL, 100 fg/mL, 10 pg/mL, 1 ng/mL, 100 ng/mL and 10 μg/mL) were tested; when testing four times, a series of Ag85B protein solutions in serum (1 fg/mL, 1 pg/mL, 1 ng/mL and 1 μg/mL) were tested; when testing three times, a series of Ag85B protein solutions in serum (1 fg/mL, 1 ng/mL and 1 μg/mL) were tested. Each test was performed from the low

to high concentration of Ag85B solution. To study the influence from serum to BSA-blocked devices, the serum was incubated onto the BSA-blocked Ab85B-SWCNT FET devices 12 times. To study the potential non-specific interaction between Ag85B and electrode, a series of Ag85B protein solutions in serum (1 fg/mL, 1 ng/mL and 1 μg/mL) were tested with BSA-blocked-SWCNT FET devices (no antibody functionalization)

5. Specificity Test

The control group for the specificity test was performed by incubating serum onto devices for 10 min, washing and then testing. SARS-CoV-2 NAg and SAg were spiked into blood serum respectively (1ng/mL). Each antigen solution was incubated onto sensor chips for 10, then washed away and tested with the same manner as stated above.

6. Detection of TB Clinical Samples

Remnant samples utilized for QuantiFeron testing at the University of Pittsburgh Medical Center, which were mainly plasma, were used for our device analysis. Clinical samples were kept frozen at -30°C before the test. 10 μL sample was incubated onto the BSA-blocked Ab85B-SWCNT devices for 10 min. Then the sample was washed away thoroughly. The FET measurement operation was the same as stated above. The protocol for collecting remnant clinical samples was reviewed and approved by the University of Pittsburgh IRB (STUDY23030154).

7. Enhancement of SWCNT Device Robustness

The deposition of SWCNT was the same as stated above. For non-covalent BSA crosslinking blocking layer incubation, the SWCNT devices were covered by a Tween20/PEG/BSA buffer (0.1% Tween 20, 4% polyethylene glycol and 1% BSA in PBS) for 1 h, which was then washed away thoroughly. Then the devices were incubated with 1% GA solution (in PBS) for 2 h for crosslinking. The excess GA solution was washed away with nanopure water. After that, the devices were incubated with 100 μL of EDC/sulfo-NHS solution (50 mM/50 mM in PBS) for 30 min to activate the carboxyl groups on BSA crosslinking layer. The excess EDC/sulfo-NHS solution was washed away with nanopure water. Then

the devices were incubated with 10 μL Ab85B (500 μg/mL in PBS) for 12 h at 4°C for the antibody functionalization. The antigen detection was the same as stated above.

For covalent BSA crosslinking blocking layer incubation (the Ab85B functionalized-crosslinked BSA-blocked-SWCNT FET devices), the devices were incubated with 100 μL of EDC/sulfo-NHS solution (50 mM/50 mM in PBS) for 30 min to activate the carboxyl groups on IsoSol-SWCNT. The excess EDC/sulfo-NHS solution was washed away with nanopure water. Then the devices were covered by a Tween20/PEG/BSA buffer (0.1% Tween 20, 4% polyethylene glycol and 1% BSA in PBS) for 1 h, which was then washed away thoroughly. Then the devices were incubated with 1% GA solution (in PBS) for 2 h for crosslinking. The excess GA solution was washed away with nanopure water. After that, the devices were incubated with 100 μL of EDC/sulfo-NHS solution (50 mM/50 mM in PBS) for 30 min to activate the carboxyl groups on BSA crosslinking layer. The excess EDC/sulfo-NHS solution was washed away with nanopure water. Then the devices were incubated with 10 μL Ab85B (500 μg/mL in PBS) for 12 h at 4°C for the antibody functionalization. To prove the improved robustness, the Ab85B-cBSA-SWCNT FET devices were exposure to a series of Ag85B solutions (in serum) from 1 fg/mL to 100 μg/mL. The FET measurement details were the same as stated above.

8. Scanning Electron Microscopy Imaging

Scanning electron microscopy (SEM) was performed with Zeiss Sigma VP Scanning Electron Microscope.

9. Atomic Force Microscopy

Atomic force microscopy (AFM) data were collected by a Bruker Multimode 8 AFM system with a Veeco Nanoscope III a controller in the tapping mode. The AFM image and height profiles were processed and obtained by Gwyddion.

10. X-ray Photoelectron Spectroscopy

X-ray photoelectron spectroscopy (XPS) data were generated on a Thermo ESCALAB 250 Xi XPS using monochromated Al Kα X-rays as the source. A 650 μm spot size was used, and the samples were charge-compensated using an electron flood gun.

11. Raman Spectroscopy

Raman spectrum was recorded with the XplorA Raman-AFM/TERS system. The radial breathing mode (RBM) region was recorded using a 785 nm (100 mW) excitation laser operating at 1% power. D and G peaks region was recorded using a 638 nm (24 mW) excitation laser operating at 1% power.

12. Fluorescence Imaging

Fluorescence images were taken with an Olympus 1X81/1X2-UCB microscope. For characterizing the amount of antibody on SWCNT devices with fluorescence imaging, the Ab85B was conjugated with FITC tag by using FITC Conjugation Kit (Fast) (ab188285; Abcam, USA) through standard protocol. Then FITC-Ab85B was functionalized onto EDC/Sulfo-NHS activated SWCNT devices by immobilization over night at 4°C. The fluorescence images were taken under excitation of 532 nm. Fluorescence images of cBSA-SWCNT devices were taken under excitation of 532 nm and 633 nm, respectively.

Principle of Debye Screening

In principle, as the negative gate voltage is applied, there is a charge separation consisting of two electrical double layers (EDLs). Specifically, cations accumulate at the interface between the gate electrode and the electrolyte while anions accumulate at the interface between the SWCNTs and the electrolyte. The width of each EDL is referred as Debye screening length (λ_D), which can be calculated by equation (S1):

$$\lambda_D = \sqrt{\frac{\varepsilon_0 \varepsilon_r k_B T}{2 N_A q^2 I}} \tag{S1}$$

where ε_0 is the vacuum permittivity, ε_r is the relative permittivity of the medium, k_B is the Boltzmann constant, T is the temperature, N_A is the Avogadro number, q is the elementary charge, and I is the ionic strength. Both EDLs will screen the rest of the ions from them. For example, for biomolecules functionalized on SWCNTs, the charged species that are beyond the Debye length will be screened.

The gating electrolytes of different concentrations have different ionic strength I, as shown in equation (S2):

$$I = \frac{1}{2} \sum C_i Z_i^2 \tag{S2}$$

where C_i is the concentration of the electrolyte and Z_i is the charge of ion.[1] The gating media used in our study were PBS solutions with varying concentrations, resulting in different ionic strengths and Debye screening length (i.e., 1×PBS: 0.7 nm, 0.1×PBS: 2.4 nm, 0.01×PBS: 7.4 nm, and 0.001×PBS: 20 nm), which may have an impact on the detection outcomes.

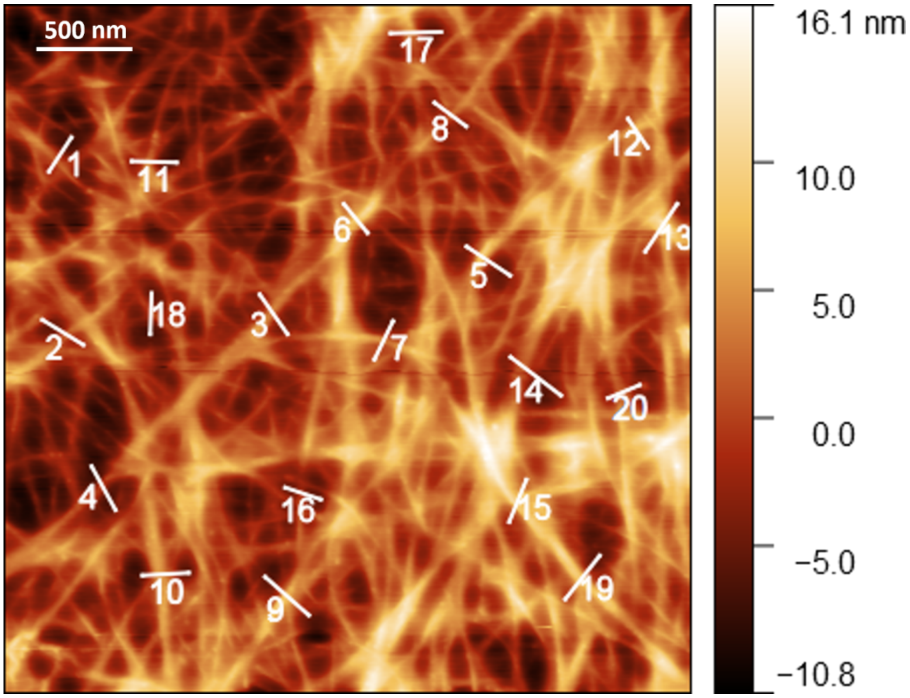

Figure S1. AFM image of a bare-SWCNT FET device with height measurement.

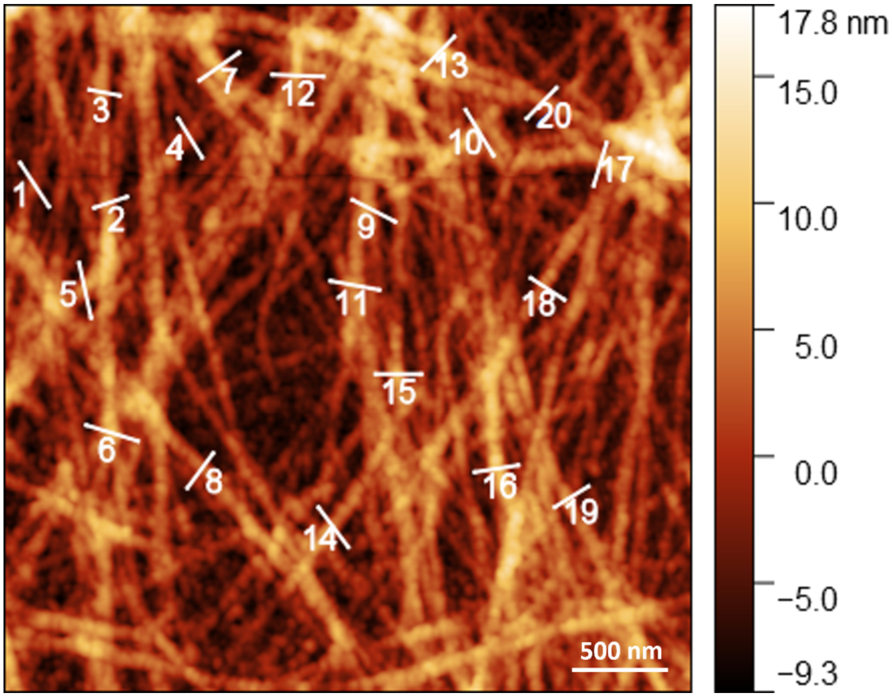

Figure S2. AFM image of an Ab85B-SWCNT FET device with height measurement.

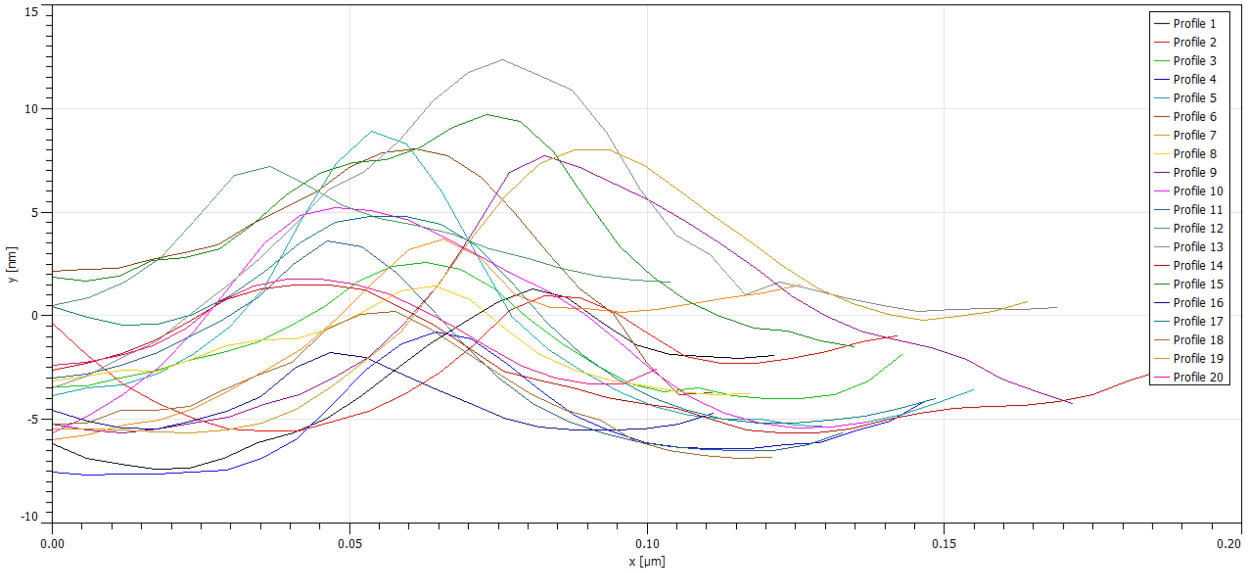

Figure S3. Height profiles (n=20) of a bare-SWCNT FET device with an average height of 9.169±3.367 nm.

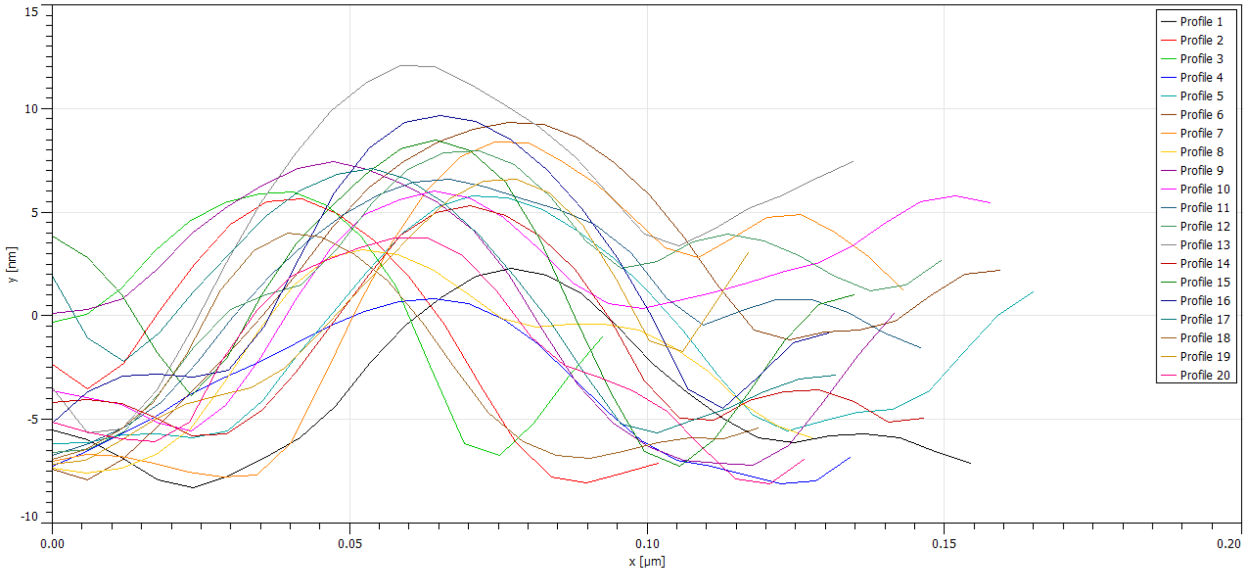

Figure S4. Height profiles (n=20) of an Ab85B-SWCNT FET device with an average height of 13.217±2.364 nm.

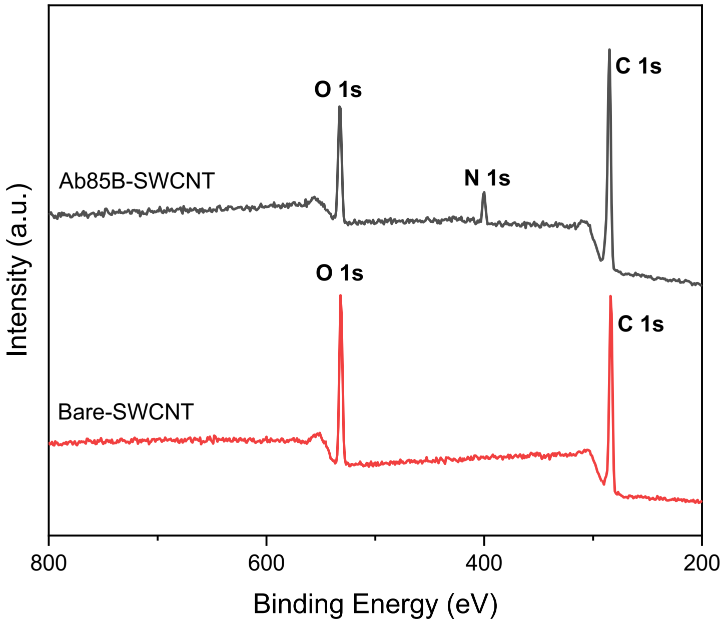

Figure S5. XPS survey of a SWCNT FET device before and after Ab85B functionalization.

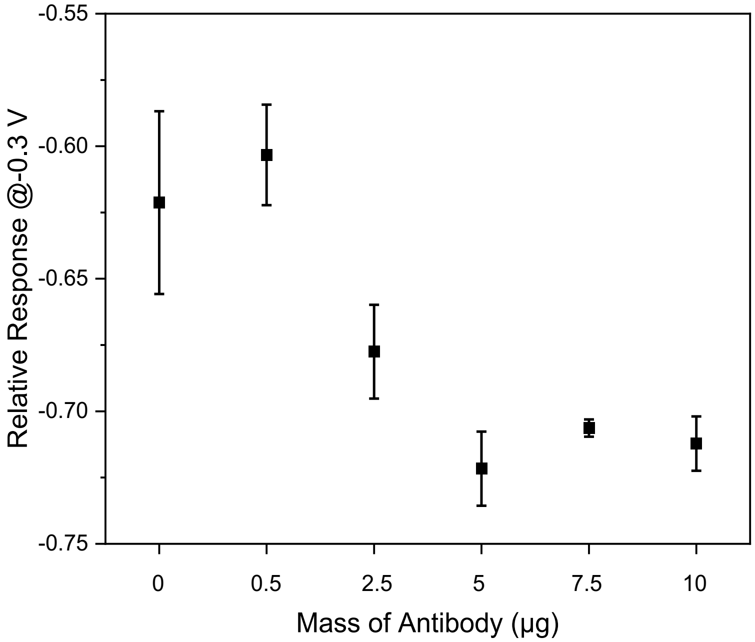

Figure S6. Calibration plot for SWCNT FET devices after antibody functionalization. All data points plotted are mean ± standard error of the mean.

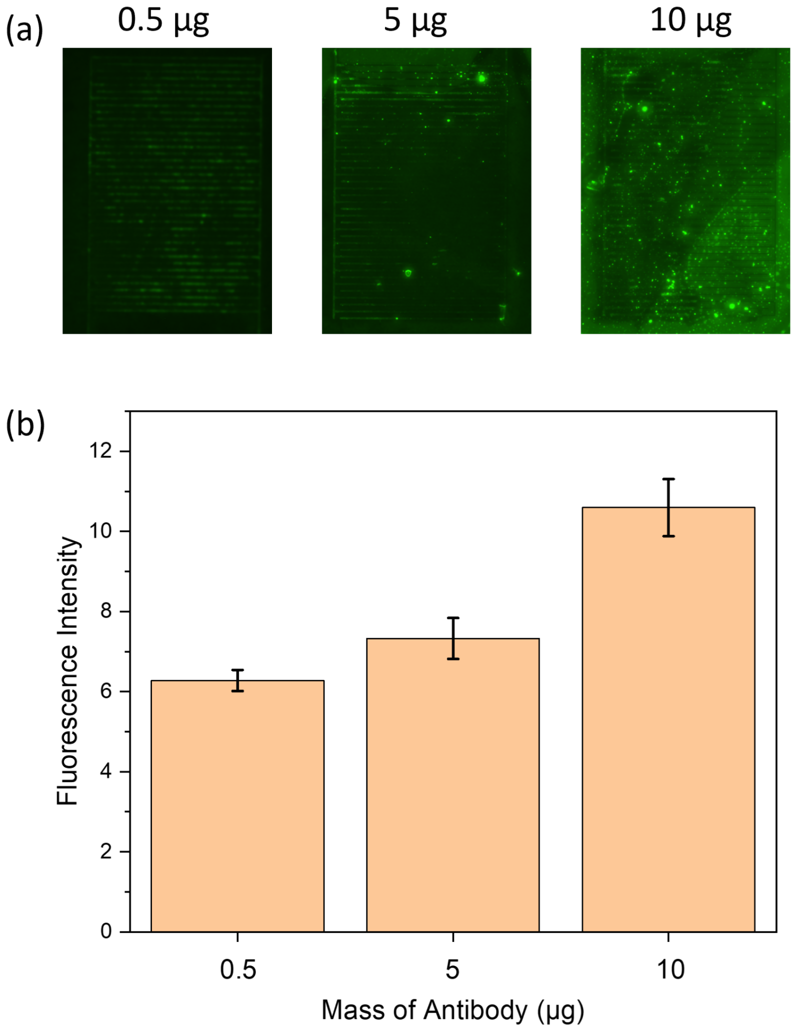

Figure S7. Fluorescence characterization of FITC-Ab85B SWCNT FET devices. (a) Fluorescence images of SWCNT devices with 0.5, 5, and 10 µg Ab85B (b) Fluorescence intensity of SWCNT devices with 0.5, 5, and 10 µg Ab85B respectively. The fluorescence intensity is analyzed with ImageJ in grayscale. All data points plotted are mean ± standard error of the mean based on eight devices.

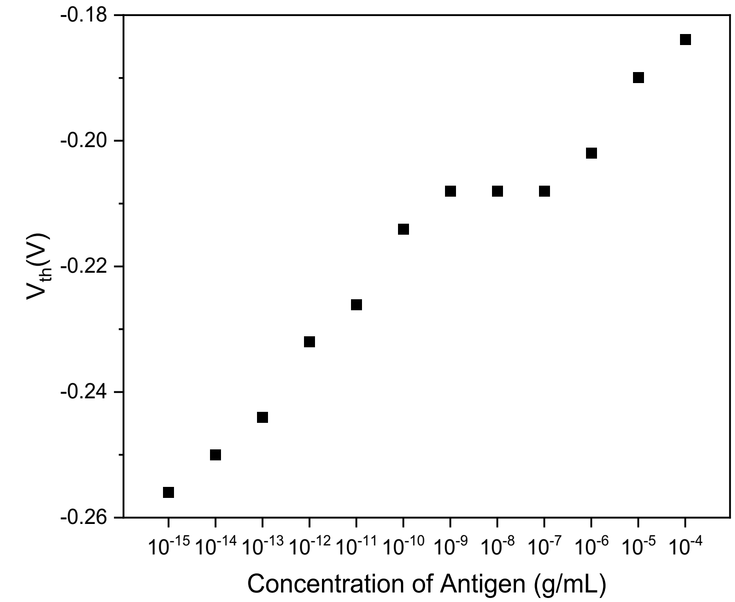

Figure S8. Threshold voltage shift of Ab85B-SWCNT FET device upon exposure to increasing concentration of Ag85B spiked in PBS.

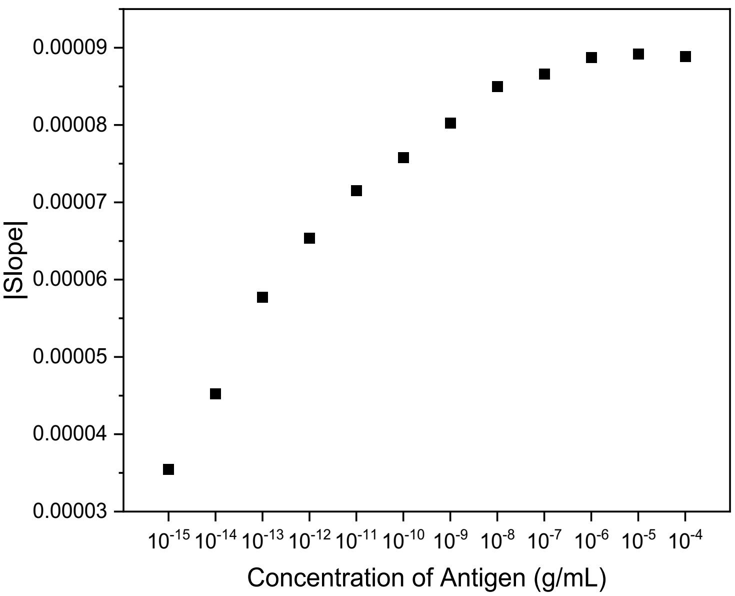

Figure S9. Characteristic curve's linear region slope of Ab85B-SWCNT FET device upon exposure to increasing concentration of Ag85B spiked in PBS.

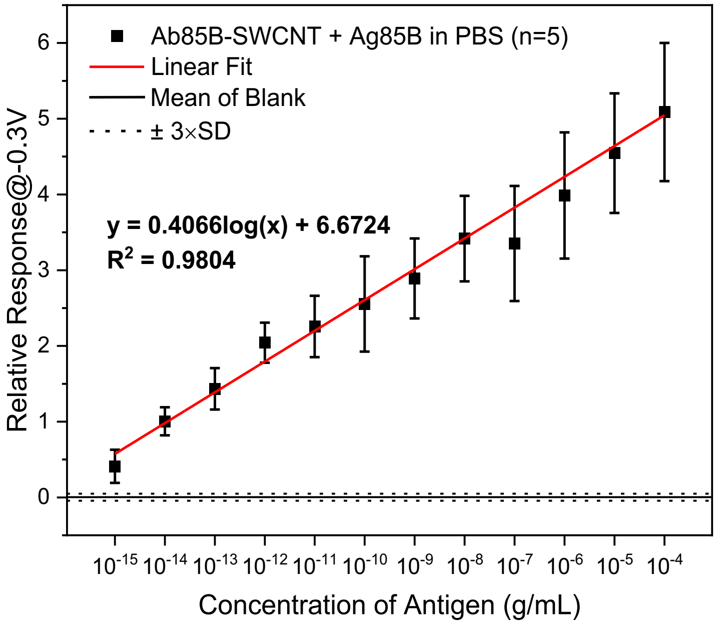

Figure S10. The calibration curve for the detection of Ag85B in PBS was linearly fit with a linear correlation coefficient of 0.9804. The mean of the source-to-drain current (I_{sd}) for blank devices at -0.3 V was 1.488 µA with a standard deviation (SD) of 0.02167 µA. The horizontal lines represent the relative response fluctuations of blank devices: mean value (0/1.488 = 0, solid line) ± 3×SD (3 × 0.02167 / 1.488 = 0.04369, dot lines). Typically, the LOD is determined by the smallest concentration of the analyte that has the device signal where the signal-to-noise ratio is greater than 3. Therefore, we plugged in three times the relative response of the SD into the regression equation 0.04369 = 0.4066*log(x) + 6.6724 and the log scale of the LOD was log(x) = –16.3. Therefore, the LOD was antilog(-16.3) = $5*10^{-17}$ or 0.05 fg/mL.

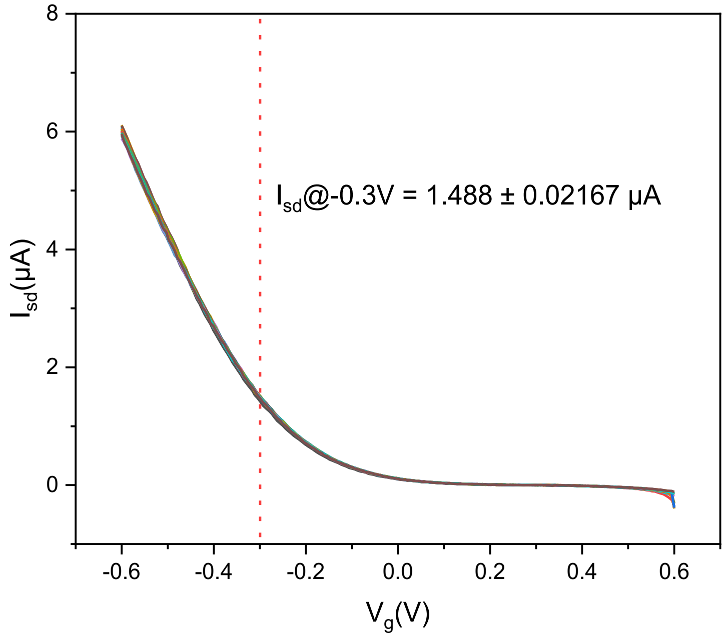

Figure S11. FET characteristic curves for Ab85B-SWCNT FET device (20 times).

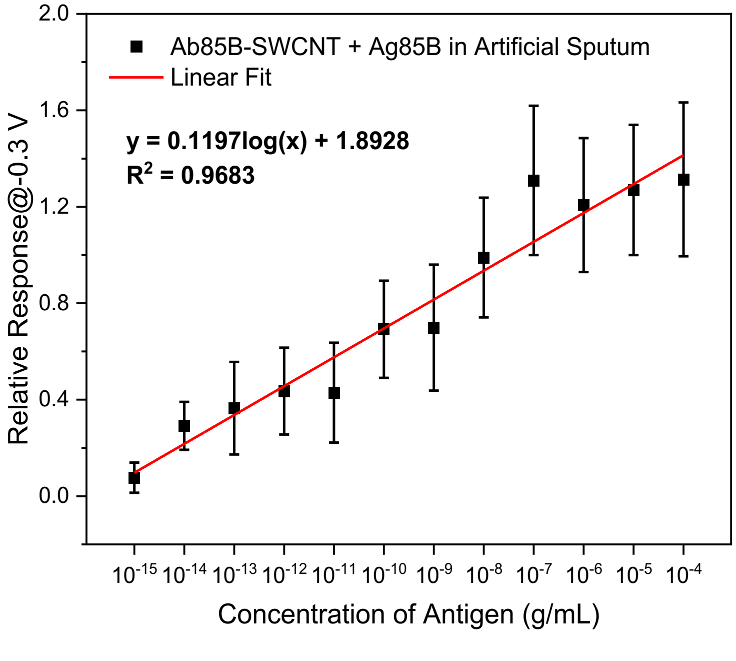

Figure S12. The calibration curve of Ag85B detection in artificial sputum was linearly fit with a linear correlation coefficient of 0.9683.

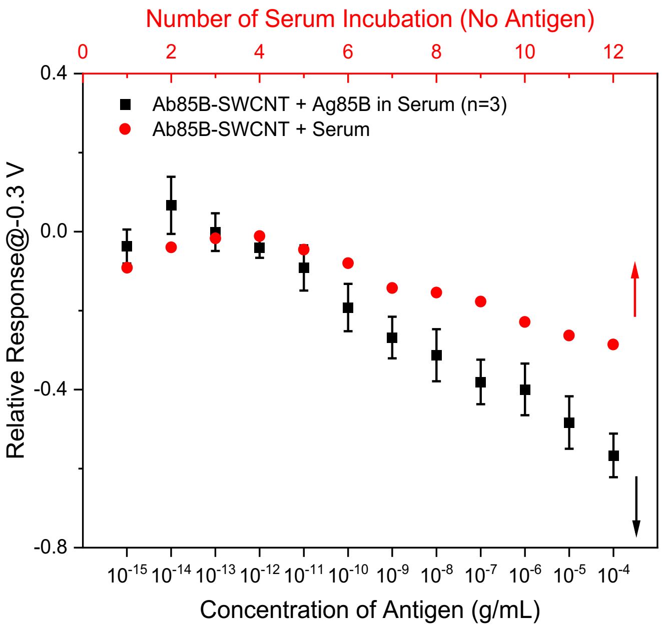

Figure S13. Calibration curve showing the detection of Ag85B in serum by Ab85B-SWCNT FET devices with error bars of mean ± standard error of the mean based on three devices and control experiment for an Ab85B-SWCNT FET device exposure to serum for 12 times. Ab85B-SWCNT FET devices were blocked by Tween 20&PEG blocking buffer.

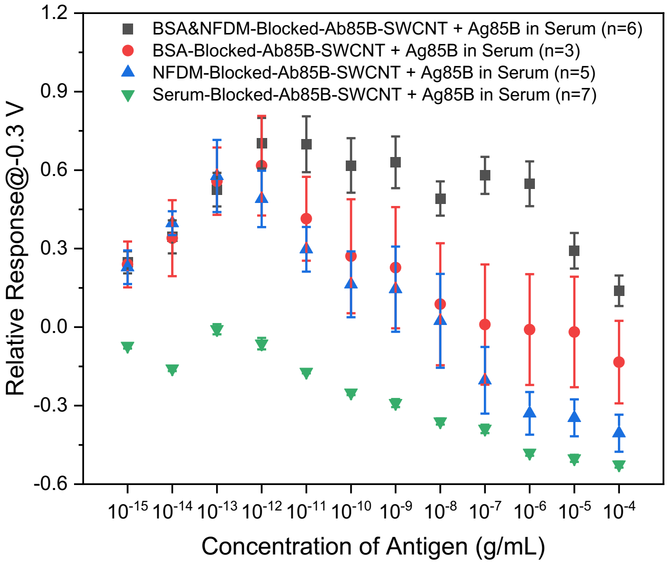

Figure S14. Calibration curve showing the detection of Ag85B in serum by BSA&NFDM-, BSA-, NFDM-, and serum-blocked Ab85B-SWCNT FET devices respectively. 12 concentrations were tested from low to high. All data points plotted in the calibration plots are mean ± standard error of the mean and the number of devices (n) used is indicated in the parenthesis.

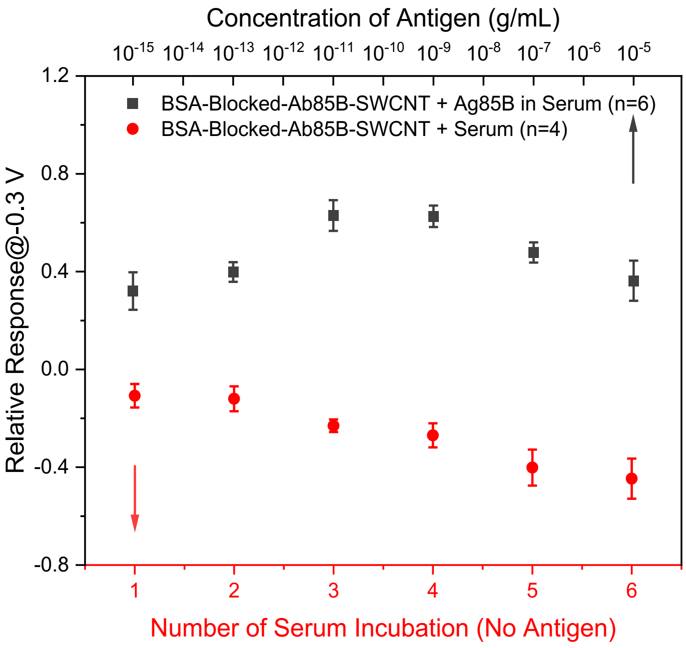

Figure S15. Calibration curve showing the detection of Ag85B in serum by BSA-blocked Ab85B-SWCNT FET devices and the effect of serum on the devices. Six concentrations were tested from low to high. All data points plotted in the calibration plots are mean ± standard error of the mean and the number of devices (n) used is indicated in the parenthesis.

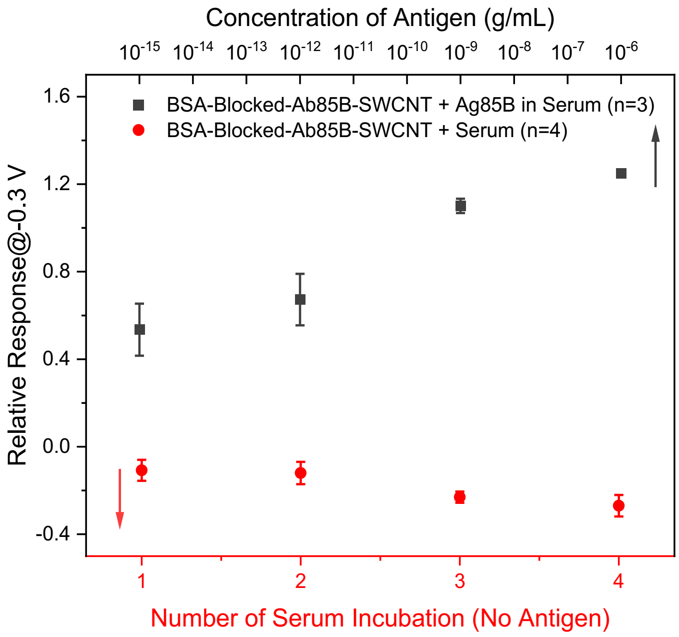

Figure S16. Calibration curve showing the detection of Ag85B in serum by BSA-blocked-Ab85B-SWCNT FET devices and the effect of serum on the devices. Four concentrations were tested from low to high concentrations. All data points plotted in the calibration plots are mean ± standard error of the mean and the number of devices (n) used is indicated in the parenthesis.

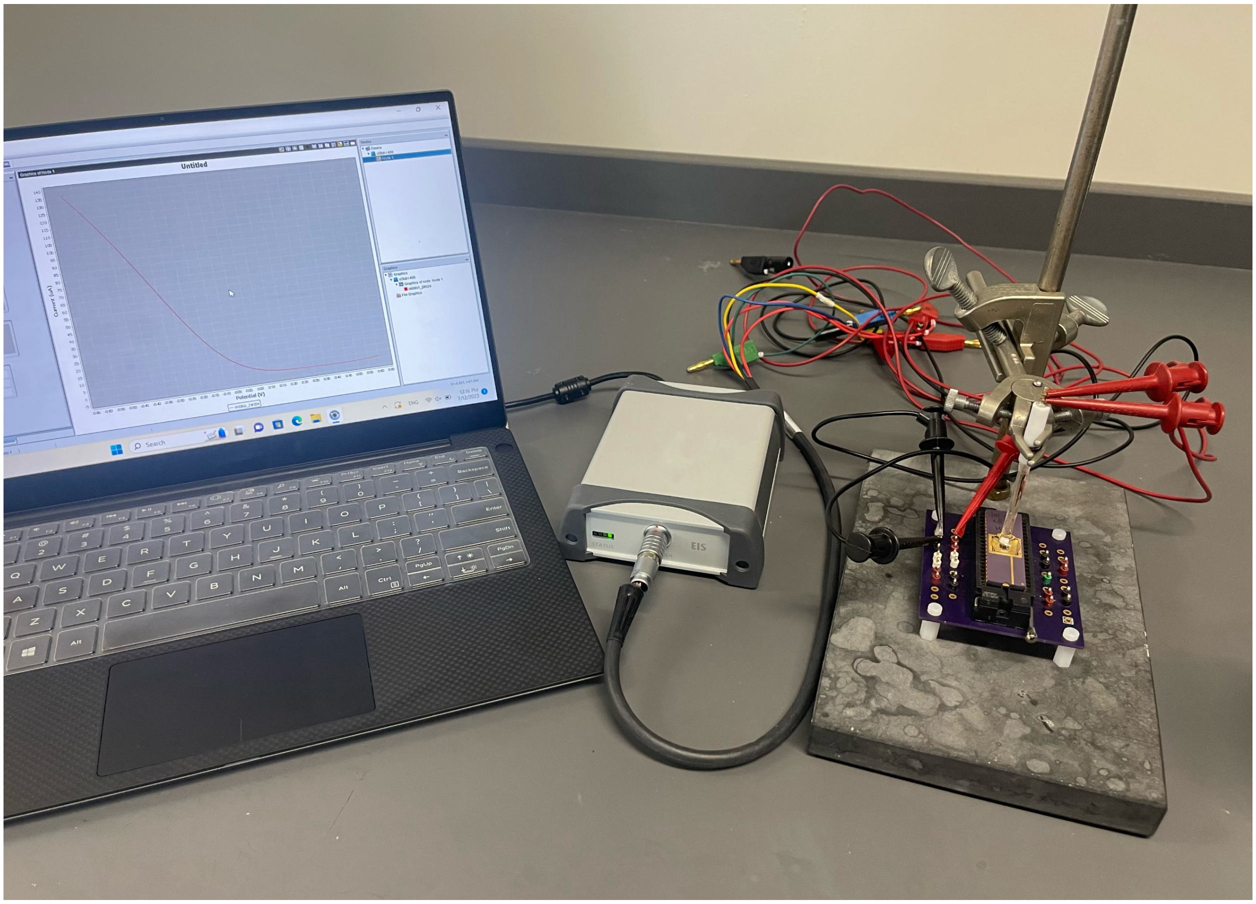

Figure S17. Photo showing the portable detection device with Metrohm potentiostat.

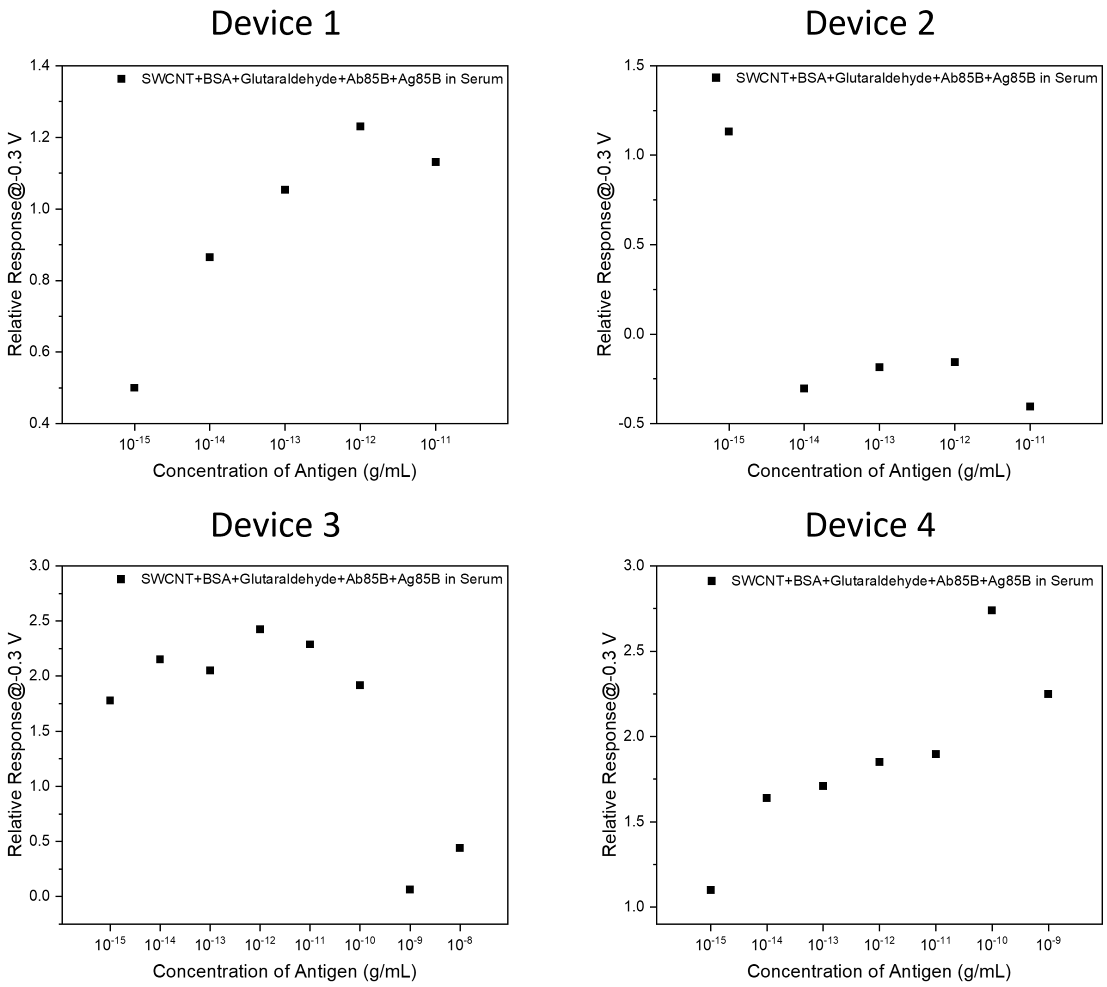

Figure S18. Calibration plot for detection of Ag85B in human peripheral blood serum with Ab85B functionalized-BSA crosslinking layer non-covalently incubated-SWCNT FET devices.

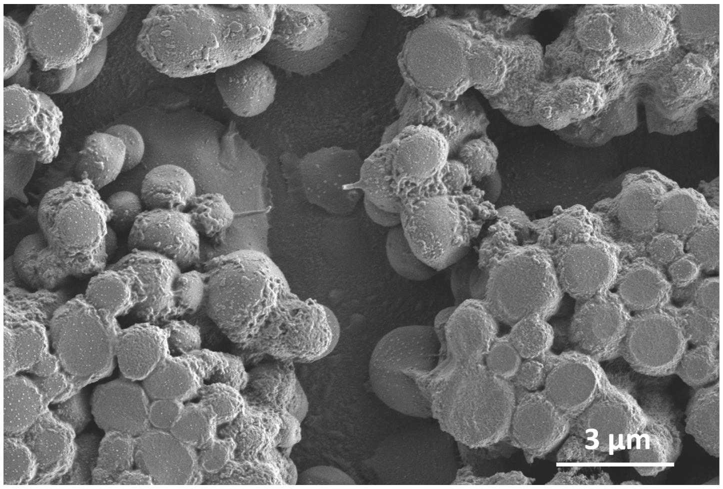

Figure S19. SEM image of a BSA-SWCNT FET device.

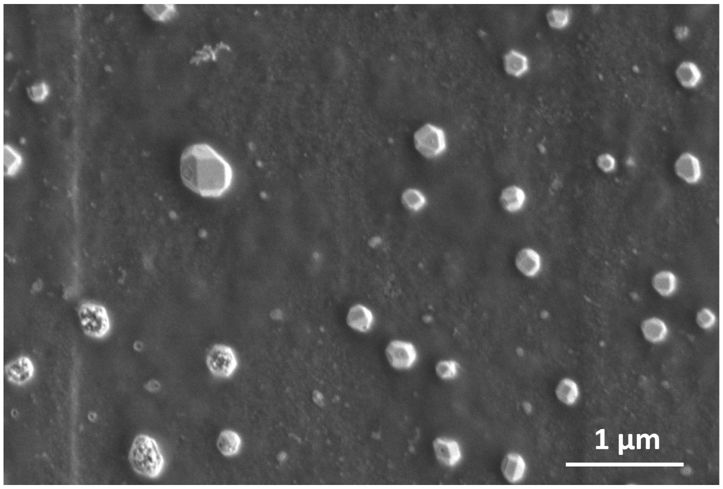

Figure S20. SEM image of a cBSA-SWCNT FET device.

a)

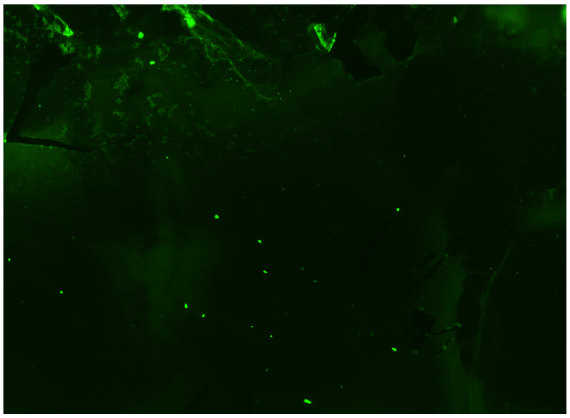

b)

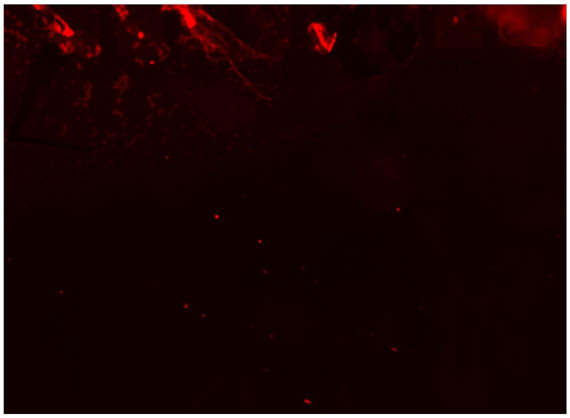

Figure S21. Fluorescence image of cBSA-SWCNT FET devices taken under excitation of a) 532 nm and b) 633 nm.

Table S1. Comparison between different technologies in TB diagnosis

Diagnosis Method	Biomarker	Detection Limit	Dynamic Range	Reference
ELISA	Antigen 85B	8 ng/mL	_	[2]
Immuno-PCR	Antigen 85B	1 fg/mL in PBS	_	[3]
E-DNA Sensor + PCR	Complementary ssDNA	0.3 fM in PBS	1fM - 100 nM	[4]
Carbon nanotube-based thin-film resistive sensor	MTB cell and MPT antigen	10 CFU/mL for MTB and 100 ng/mL of MPT64 in tongue swab samples; 100 CFU/mL for MTB and 1 ng/mL for MPT64 in sputa	_	[5]
CdSe/ZnS QD/SiNP Immunosensor	CFP10-ESAT6	0.15 ng/mL in PBS	40 - 100 ng/mL	[6]
Graphene FET biosensor	Interferon-gamma	82.7 pM in PBS	_	[7]
Silicon nitride ion sensitive FET biosensor	Ag85B	0.12 µg/mL in PBS	0.12 - 1 µg/ml	[8]
SiNW FET biosensor	Ag85B	0.01 fg/mL in PBS	1 fg/mL - 100 µg/mL	[9]
SWCNT FET biosensor	Ag85B	0.05 fg/mL in PBS and 1 fg/mL in artificial sputum and blood serum	1 fg/mL - 100 µg/mL	This work

References

(1) Purwidyantri, A.; Domingues, T.; Borme, J.; Guerreiro, J. R.; Ipatov, A.; Abreu, C. M.; Martins, M.; Alpuim, P.; Prado, M. Influence of the electrolyte salt concentration on DNA detection with graphene transistors. *Biosensors* **2021**, *11* (1), 24.

(2) Phunpae, P.; Chanwong, S.; Tayapiwatana, C.; Apiratmateekul, N.; Makeudom, A.; Kasinrerk, W. Rapid diagnosis of tuberculosis by identification of Antigen 85 in mycobacterial culture system. *Diagn. Microbiol. Infect. Dis.* **2014**, *78* (3), 242-248.

(3) Singh, N.; Sreenivas, V.; Gupta, K. B.; Chaudhary, A.; Mittal, A.; Varma-Basil, M.; Prasad, R.; Gakhar, S. K.; Khuller, G. K.; Mehta, P. K. Diagnosis of pulmonary and extrapulmonary tuberculosis based on detection of mycobacterial antigen 85B by immuno-PCR. *Diagn. Microbiol. Infect. Dis.* **2015**, *83* (4), 359-364.

(4) Miodek, A.; Mejri, N.; Gomgnimbou, M.; Sola, C.; Korri-Youssoufi, H. E-DNA sensor of Mycobacterium tuberculosis based on electrochemical assembly of nanomaterials (MWCNTs/PPy/PAMAM). *Anal. Chem.* **2015**, *87* (18), 9257-9264.

(5) Kahng, S.-J.; Soelberg, S. D.; Fondjo, F.; Kim, J.-H.; Furlong, C. E.; Chung, J.-H. Carbon nanotube-based thin-film resistive sensor for point-of-care screening of tuberculosis. *Biomed. Microdevices* **2020**, *22*, 1-10.

(6) Mohd Bakhori, N.; Yusof, N. A.; Abdullah, J.; Wasoh, H.; Ab Rahman, S. K.; Abd Rahman, S. F. Surface enhanced CdSe/ZnS QD/SiNP electrochemical immunosensor for the detection of Mycobacterium tuberculosis by combination of CFP10-ESAT6 for better diagnostic specificity. *Materials* **2019**, *13* (1), 149.

(7) Farid, S.; Meshik, X.; Choi, M.; Mukherjee, S.; Lan, Y.; Parikh, D.; Poduri, S.; Baterdene, U.; Huang, C.-E.; Wang, Y. Y. Detection of Interferon gamma using graphene and aptamer based FET-like electrochemical biosensor. *Biosens. Bioelectron.* **2015**, *71*, 294-299.

(8) Saengdee, P.; Chaisriratanakul, W.; Bunjongpru, W.; Sripumkhai, W.; Srisuwan, A.; Hruanun, C.; Poyai, A.; Phunpae, P.; Pata, S.; Jeamsaksiri, W.; Kasinreak, W.; Promptmas, C. A silicon nitride ISFET based immunosensor for Ag85B detection of tuberculosis. *Analyst* **2016**, *141* (20), 5767-5775.

(9) Ma, J.; Du, M.; Wang, C.; Xie, X.; Wang, H.; Li, T.; Chen, S.; Zhang, L.; Mao, S.; Zhou, X.; Wu, M. Rapid and sensitive detection of mycobacterium tuberculosis by an enhanced nanobiosensor. *ACS Sens.* **2021**, *6* (9), 3367-3376.
